# Convenient synthesis and delivery of a megabase-scale designer accessory chromosome empower biosynthetic capacity

**DOI:** 10.1038/s41422-024-00934-3

**Published:** 2024-02-08

**Authors:** Yuan Ma, Shuxin Su, Zongheng Fu, Chu Zhou, Bin Qiao, Yi Wu, Ying-Jin Yuan

**Affiliations:** 1 Frontiers Science Center for Synthetic Biology and Key Laboratory of Systems Bioengineering (Ministry of Education), School of Chemical Engineering and Technology, Tianjin University, Tianjin, China; 2 Frontiers Research Institute for Synthetic Biology, Tianjin University, Tianjin, China

**Keywords:** Biological techniques, Molecular biology

## Abstract

Synthetic biology confers new functions to hosts by introducing exogenous genetic elements, yet rebuilding complex traits that are based on large-scale genetic information remains challenging. Here, we developed a CRISPR/Cas9-mediated haploidization method that bypasses the natural process of meiosis. Based on the programmed haploidization in yeast, we further developed an easy-to-use method designated HAnDy (Haploidization-based DNA Assembly and Delivery in yeast) that enables efficient assembly and delivery of large DNA, with no need for any fussy in vitro manipulations. Using HAnDy, a de novo designed 1.024 Mb synthetic accessory chromosome (synAC) encoding 542 exogenous genes was parallelly assembled and then directly transferred to six phylogenetically diverse yeasts. The synAC significantly promotes hosts’ adaptations and increases the scope of the metabolic network, which allows the emergence of valuable compounds. Our approach should facilitate the assembly and delivery of large-scale DNA for expanding and deciphering complex biological functions.

## Introduction

Acquiring functional genetic elements to expand the host genome is critical in natural evolution^1^ and bioengineering.^2^ In nature, the acquisition of exogenous genetic materials through horizontal gene transfer (HGT) or genome introgression has occurred in many organisms, which may facilitate hosts’ environmental adaptation.^3–5^ Recent studies have reported that genome expansion can be achieved at megabase scale.^6,7^ An archaea *Methanoperedens* spp. contains a ~1 Mb extrachromosomal circular DNA called ‘Borgs’, with potential to expand metabolic capacity and increase the ability of methane oxidation.^7^ In bioengineering, introducing heterogeneous genetic materials from multiple species into a single organism to reconstruct biological processes is a standard practice.^8^ For example, dozens of heterogeneous genes have been introduced into yeast from plants, bacteria, and other fungi to produce alkaloids, terpenoids, and other natural products.^9–12^ It is reasonable that reconstructing complex biological functions often requires introduction of large-scale genetic materials.^13^ Thus, methods of convenient assembly and delivery of large-scale DNA are essential.^14^

For large-scale DNA assembly, *Saccharomyces cerevisiae* is the primary choice due to its efficient homologous recombination capability.^15,16^ Two major strategies have been developed to achieve megabase-scale DNA assembly in yeast: one-step large-scale DNA assembly^17,18^ and iterative assembly of relatively small fragments.^19–21^ Protoplast-based transformation associated recombination (TAR) assembly enabled one-step transformation of eleven fragments with the length of ~100 kb into *S. cerevisiae* and assembly of a *Mycoplasma mycoides* genome up to 1.08 Mb. However, the assembly process is complicated because it requires complex in vitro manipulation procedures to isolate and purify high-quality 100 kb fragments from yeast. In addition, the assembly efficiency is low because many large fragments need to be introduced into a cell simultaneously.^17,20^ To avoid the complexity of large-scale DNA manipulation, large-scale DNA can be split into several small fragments for iterative assembly.^16^ However, this approach is time-consuming, especially for assembly of the megabase-scale DNA. For example, 18 rounds of the SwAP-In cycle were required to construct the synthetic yeast ChrX (~700 kb) in a stepwise manner in the Sc 2.0 project.^19^ Although other iterative assembly methods have been reported, they often require complicated processes (such as meiosis and protoplast fusion) for introducing the large-scale DNA into the assembly host.^20–22^ In addition, the delivery of large-scale DNA from the assembly host into diverse recipients is imperative for expanding the scope of application in synthetic genomics.^14^ However, the current methods are inefficient because the shearing during the isolation process may affect the integrity of the delivered DNA, especially for DNA larger than 1 Mb.^16^ Therefore, the main challenge in the assembly and delivery of large-scale DNA is the efficiency of introducing intact large-scale DNA into cells, both for assembly in host cells and delivery into recipient cells.

Here we established an easy-to-use method designated HAnDy (Haploidization-based DNA Assembly and Delivery in yeast), enabling efficient assembly and delivery of a de novo designed 1.024 Mb synthetic accessory chromosome (synAC). The synAC contains 542 accessory genes from a collection of 1011 *S. cerevisiae* isolates, providing a comprehensive evolutionary driving force for host adaptation. Moreover, the synAC dramatically improved the biosynthetic capacity, allowing the production of several natural products that are difficult to synthesize heterologously.

## Results

### De novo design of a megabase synAC

**Fig. 1:**
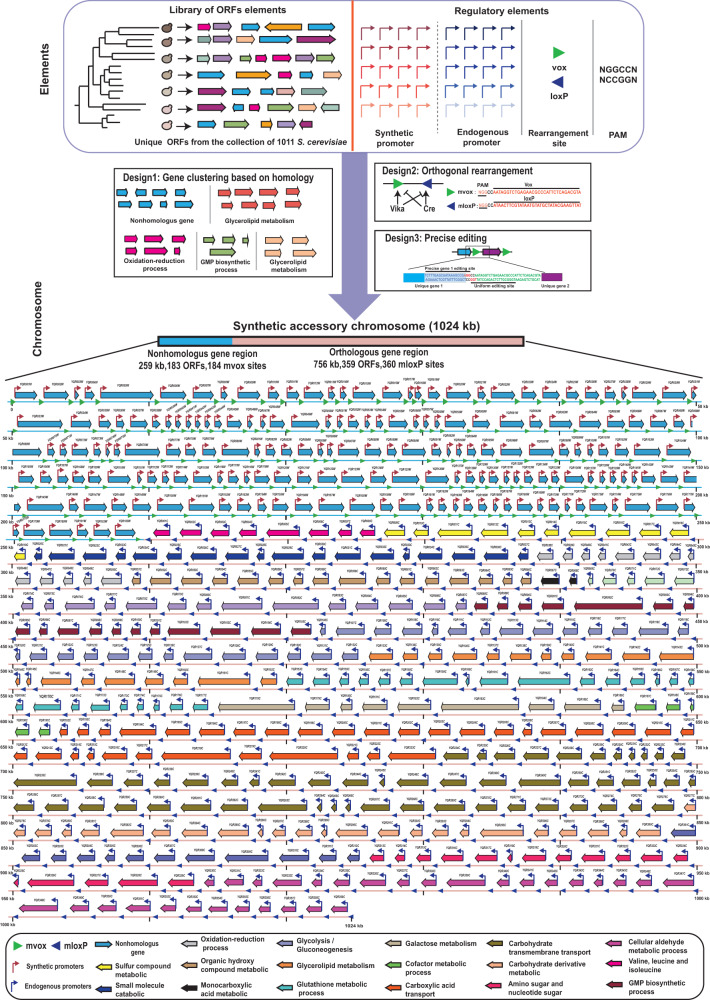
Schematic diagram for the de novo design of the synAC. The top box represents basic elements of the synAC. The middle boxes illustrate the three design principles for synAC. The diagram at the bottom shows the detailed structure of synAC.

Large-scale population genomic surveys categorize genes into the core and accessory genes, the former being present in all members and the latter in only a subset of strains.^23^ To demonstrate the capacity of accessory genes in functional diversity, we designed a synAC by introducing large-scale accessory genes from different *S. cerevisiae* isolates. Several principles of genome design have been defined in synthetic genomics efforts to recode^24,25^ and reorganize^26,27^ genomes, which often rely on the native genomes as templates. There are no native templates for the design of extrachromosomal chromosome, and therefore we need to de novo design the synthetic chromosome from individual accessory genes and regulatory elements. To confer genetic flexibility and modularity to the synAC, allowing for a rapid response to designer bugs and facilitating characterization of the relationship between genotypes and phenotypes, we defined the following three rules for the design of the synAC: first, clustering of functionally related genes; second, establishment of an orthogonal chromosomal rearrangement system; and third, introduction of a precise editing system (Fig. 1). As a pilot, we designed a megabase-scale synAC following these three rules to expand the genome of *S. cerevisiae*.

We utilized the genomic data from the collection of 1011 *S. cerevisiae* isolates, identifying 1715 accessory genes that are variable within the population but absent in *S. cerevisiae* S288C.^23^ According to the homology analysis of S288C, these genes could be divided into two types: orthologous and non-homologous genes. The orthologous genes may have diverged via the accumulation of mutations during yeast evolution, and these genes may be instrumental in enhancing host adaptations.^23^ Non-homologous genes may be derived from HGT or genome introgression and may confer novel functions to the host.^6,28^ To facilitate the analysis of the functions of non-homologous and orthologous genes in yeast adaptation, the synAC was segmented into non-homologous and orthologous gene regions (Fig. 1). For the orthologous genes, a total of 359 orthologous ORFs were identified in the Yeast 8.0 metabolic database,^29^ and we further clustered the functionally related genes by Metascape (Supplementary information, Fig. S1).^30^ For the non-homologous genes, we selected 183 genes which are not present in S288C but present in more than five strains among the 1011 isolates. The functionality of the synAC relies on the expression of accessory genes. However, characterizing the expression of these genes in a vast number of the original strains is rather laborious. To ensure the expression of the exogenous genes, we employed synthetic and endogenous promoters for non-homologous and orthologous genes, respectively. Synthetic promoters were selected from a library of 100 million yeast promoters,^31^ while the promoters for the orthologous genes were derived from their orthologs in S288C (Supplementary information, Data S1).

To facilitate characterization of the relationship between genotypes and phenotypes and confer synthetic chromosome the ability to evolve, we introduced two genetic manipulation systems in the synAC: an orthogonal random rearrangement system and a precise gene editing system. Learning from the Sc 2.0 project,^32,33^ for the design of the orthogonal rearrangement system, we introduced numerous orthogonal rearrangement sites (loxP and vox) into the synAC. The vox sites were placed upstream of each non-homologous gene and the loxP sites were placed at equivalent positions in the region of orthologous genes. The Cre/loxP and Vika/vox represent a set of orthogonal rearrangement system,^34,35^ whereby induction of Cre or Vika recombinase enables orthogonal rearrangement of the synAC in orthologous gene region and non-homologous gene region, respectively (Supplementary information, Fig. S2). For the design of the precise editing system, we introduced numerous “GGCC” sequences after the terminators of each accessory gene which can serve as specific single guide RNA (sgRNA) target sites for customized rearrangement of the synAC by CRISPR/Cas9 (Fig. 1).^36^ Moreover, the inserted “GGCC” sequences combined with the adjacent loxP and vox sites can be uniform sgRNA target sites that may serve as a potential biocontainment system to prevent the escape of the synAC into the natural environment by CRISPR/Cas9-mediated multiplex cutting.

Following these rules, we de novo designed a 1.024 Mb synAC encoding 542 exogenous genes (Fig. 1; Supplementary information, Data S1), consisting of 183 non-homologous genes and 359 orthologous genes. To confer genetic flexibility, we incorporated 184 vox sites in the non-homologous gene region, 360 loxP sites in the orthologous gene region, and 544 “GGCC” sequences before all the rearrangement sites.

### CRISPR/Cas9-mediated haploidization that bypasses meiosis

**Fig. 2:**
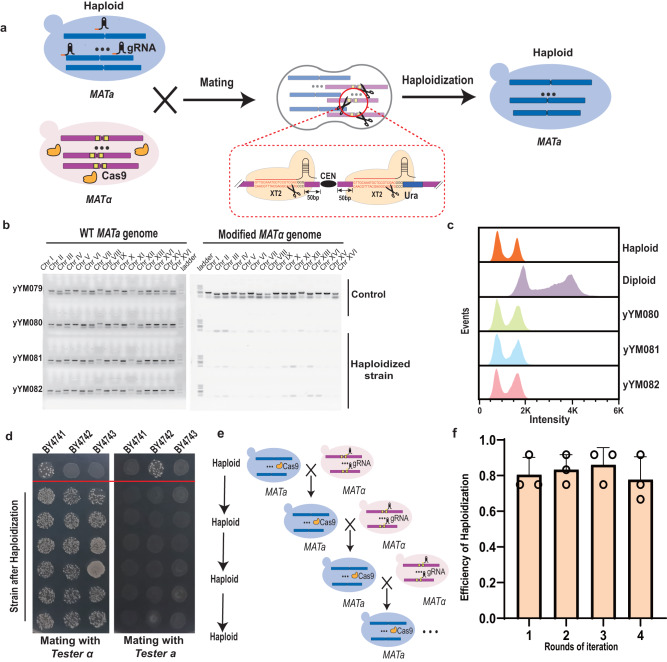
CRISPR/Cas9-mediated haploidization that bypasses meiosis. **a** Schematic illustration of CRISPR/Cas9-mediated genome elimination. **b** PCRTag analysis of the haploidized strains. Sixteen PCRTags for the WT BY4741 (*MATa*) genome and 16 PCRTags for the modified BY4742 (*MATα*) genome were used for verification. The diploid strain yYM079 generated after mating of WT BY4741 and BY4742^XT2-URA^ without sgRNA was used as control. **c** Analysis of the DNA content after haploidization by flow cytometry. Three experimental strains (yYM080, yYM081 and yYM082) were analyzed, and a haploid strain BY4741 and a diploid strain BY4743 served as controls. **d** Evaluation of mating ability of the progeny after haploidization by *Tester* a and *Tester* α. **e** Schematic illustration of multiple rounds of haploidization. First, BY4741 (blue) carrying the Cas9 plasmid was mated with BY4742^XT2-URA^ (pink) carrying the sgRNA plasmid, completing the first round of haploidization. Then the haploid strain was mated with BY4742^XT2-URA^ to initiate the second round of haploidization. **f** Efficiency of haploidization in multiple rounds. A total of 12 strains were tested by flow cytometry for each replicate. Data are shown as mean ± SD (*n* = 3 three biological replicates).

Haploidization is a biological process of halving the chromosomal content of a cell and producing a haploid cell from a diploid cell. In the normal life cycle of *S. cerevisiae*, a *MATa* haploid cell mates with a *MATα* haploid cell to form a diploid cell, followed by meiosis to generate haploid spores. Previously, we found that creating a single double-strand break (DSB) using CRISPR/Cas9 near the centromere of *S. cerevisiae* can cause the loss of the entire chromosome in diploid strains.^37^ What if creating DSBs at all 16 centrosomes in *S. cerevisiae* via CRISPR/Cas9, could it selectively eliminate half of the genome in diploid yeast? To demonstrate the potential of CRISPR/Cas9-mediated haploidization, we constructed a strain BY4742^XT2-URA^, in which a Cas9 uniform target site XT2 and a *URA3* gene were inserted adjacent to the centromere of all 16 chromosomes (Fig. 2a; Supplementary information, Fig. S3). The CRISPR/Cas9-induced DSBs at all centromeres were triggered by co-culture of the haploid strains BY4741 and BY4742^XT2-URA^, which harbor guide RNA (gRNA) and Cas9 plasmids, respectively. Subsequently, the mating cells were spread onto the selective medium containing 5-fluoroorotic acid (5-FOA) to counter-select any remaining *URA3*-modified chromosomes. The counter-selected colonies were tested by PCR using specific PCRTags, and the results showed that all 16 PCRTags against the BY4742^XT2-URA^ were lost in the tested strains (Fig. 2b). In addition, DNA content measurement and whole-genome sequencing of three strains (yYM080, yYM081, and yYM082) after haploidization indicated that the entire genome of BY4742^XT2-URA^ was eliminated (Fig. 2c; Supplementary information, Fig. S4). To evaluate the mating ability of strains after haploidization, 18 colonies were randomly selected for mating with the tester strains. Similar to BY4741, all haploidized strains were able to mate again with *Tester α* but could not mate with *Tester a* (Fig. 2d), which confirms that the progeny after chromosome elimination can mate again. To demonstrate the ability of CRISPR/Cas9-mediated genome elimination in multiple rounds of haploidization, we initiated four rounds of haploidization cycle in BY4741 and BY4742^XT2-URA^ (Fig. 2e). For each round, a total of 36 colonies were analyzed by flow cytometry, and the results showed that efficiency of haploidization is ~80% (Fig. 2f). We conclude that inducing DSBs at the centromeres of all chromosomes in *S. cerevisiae* via CRISPR/Cas9 could efficiently eliminate one set of the genome in diploid yeast. More importantly, the method of haploidization by genome elimination endows yeast with the ability to undergo sustained mating bypassing the process of meiosis.

### Rationale of sustained DNA assembly via haploidization in yeast

**Fig. 3:**
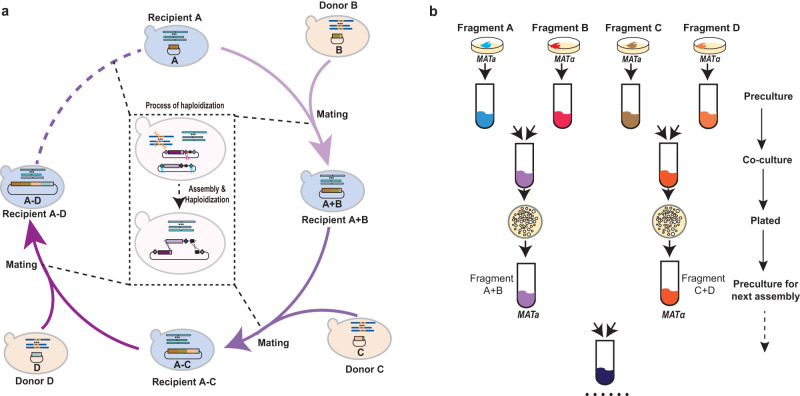
Rationale of sustained DNA assembly via haploidization in yeast. **a** Mechanism for haploidization-based sustained DNA assembly. Once BY4741 (*MATa*) mates with BY4742^XT2-Ura^ (*MATα*), two episomal DNAs could be introduced into one cell. With assistance of CRISPR/Cas9 system, the assembly of episomal DNA and haploidization of genome can be completed. Then the assembled strains can immediately initiate the next round of assembly by mating with another strain. See Supplementary information, Fig. S5 for a detailed mechanism. **b** Experimental workflow for haploidization-based sustained DNA assembly in yeast.

*S. cerevisiae* is the primary choice for large-scale DNA assembly; however, the main challenge in assembling the large-scale DNA is the low efficiency of introducing the intact large-scale DNA into assembly host cells.^38^ We have demonstrated that CRISPR/Cas9-mediated haploidization enables yeasts to mate consecutively, through which the episomal DNA in yeast cells with opposite mating types can be continuously and efficiently introduced into one cell. Therefore, we proposed nesting of the DNA assembly process into the programmed haploidization cycle to establish a large-scale DNA iterative assembly method (Fig. 3a). Briefly, two sets of episomal DNA that are pre-constructed in the yeasts with opposite mating types can be introduced into one cell during mating. With the assistance of the CRISPR/Cas9 system, the donor DNA is assembled into the linearized recipient plasmid through yeast homologous recombination, and the haploidization is completed by programmed elimination of one set of genomes (Supplementary information, Fig. S5). More importantly, the haploidized strain can mate with another donor strain immediately to initiate the next round of assembly (Fig. 3b). Thus, this approach enables convenient iterative assembly of large-scale DNA without any inefficient manipulation.

### Assembly of the megabase synAC by programmed haploidization

**Fig. 4:**
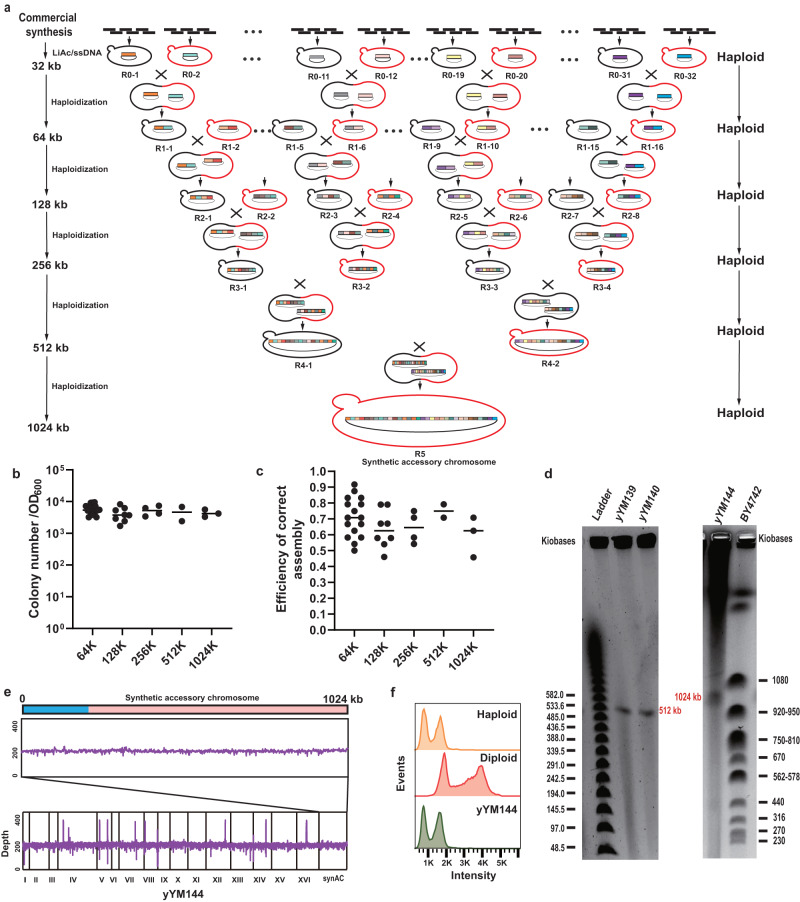
Assembly of the megabase synAC by programmed haploidization. **a** Parallel workflow for assembly of the megabase synAC by programmed haploidization. Black and red lines indicate BY4742^XT2-URA^ and BY4741^XT1-FCY^, respectively. **b** Number of colonies in each assembly. The data were obtained from the screening plate per 10^7^ cells from the mating medium. Data points were collected from independent assembly reactions. **c** Assembly accuracy in the process of megabase-scale assembly. The assembly accuracy was determined by PCR validations of assembly junctions. Data points were collected from independent assembly reactions. **d** PFGE validations of the assembled constructs. The assembled circular DNA constructs were isolated from yeast in low-melting agarose plugs and subjected to PFGE analysis by *I-SceI*. Using New England Biolabs (NEB) Lambda PFG Ladder (left) and WT *S. cerevisiae* BY4742 chromosomes (right) as makers to validate 512 kb and 1024 kb constructs, respectively. The correct sizes of the assembled DNA constructs are indicated beside the bands. **e** Sequencing read depths of all 16 chromosomes and the synAC in the final strain yYM144. **f** DNA content of the final strain yYM144 was analyzed by flow cytometry. A haploid strain BY4741 and a diploid strain BY4743 served as controls.

To determine the capability of DNA assembly by programmed haploidization, we set up a completely in vivo iterative workflow for assembly of the megabase accessory chromosome from 180 commercially synthesized small fragments (~6 kb) as diagrammed in Fig. 4a. First, sets of five or six adjacent fragments were introduced into *S. cerevisiae* to construct 32 initial R0-series fragments (~32 kb) by TAR assembly, among which two adjacent R0-series fragments were constructed in strains with opposite mating types (Supplementary information, Data S2). Following mating of two adjacent strains harboring R0-series fragments, 16 R1-series assemblies (~64 kb) were generated, and then mating of the haploidized R1-series strains produced 8 R2-series assemblies (~128 kb) and so forth. After five rounds of programmed haploidization-based assembly, the megabase synAC was completely assembled in yeast (Supplementary information, Data S3).

For each round of assembly, we easily obtained > 10^3^ colonies in the selective medium (Fig. 4b; Supplementary information, Fig. S6). To determine the accuracy of assembly, 24 colonies were randomly selected and PCR validations of assembly junctions were performed for each assembly. The results show that the accuracy of all assembly is ~60% (Fig. 4c; Supplementary information, Fig. S6), which means that on average, one correct assembly can be identified for randomly selected two colonies. We further validated the size of assembled constructs by pulsed-field gel electrophoresis (PFGE). The corresponding bands in the PFGE gel present the correct assembly of the constructs and provide evidence that the Mb-size DNA could be assembled by programmed haploidization (Fig. 4d). In addition, we further verified the sequential integrality of the synAC in yYM144 strain by PCR and whole-genome sequencing (Fig. 4e; Supplementary information, Figs. S7, S8). Moreover, flow cytometry analysis shows that the final strain remains haploid after five rounds of haploidization, and maintains the ability to undergo further assembly of larger DNA (Fig. 4f), and synAC exhibits the genetic stability after 100 generations of passages (Supplementary information, Fig. S9). In the assembly process of the megabase synAC, the number of colonies and the assembly accuracy are relatively stable, indicating that this method may be size-independent. More importantly, the assembly process only relies on spontaneous mating and programmed haploidization between two yeasts to complete the Mb-size assembly without any complex and time-consuming operations, demonstrating that this method is easy to use.

### Delivery of the megabase synAC by programmed haploidization

**Fig. 5:**
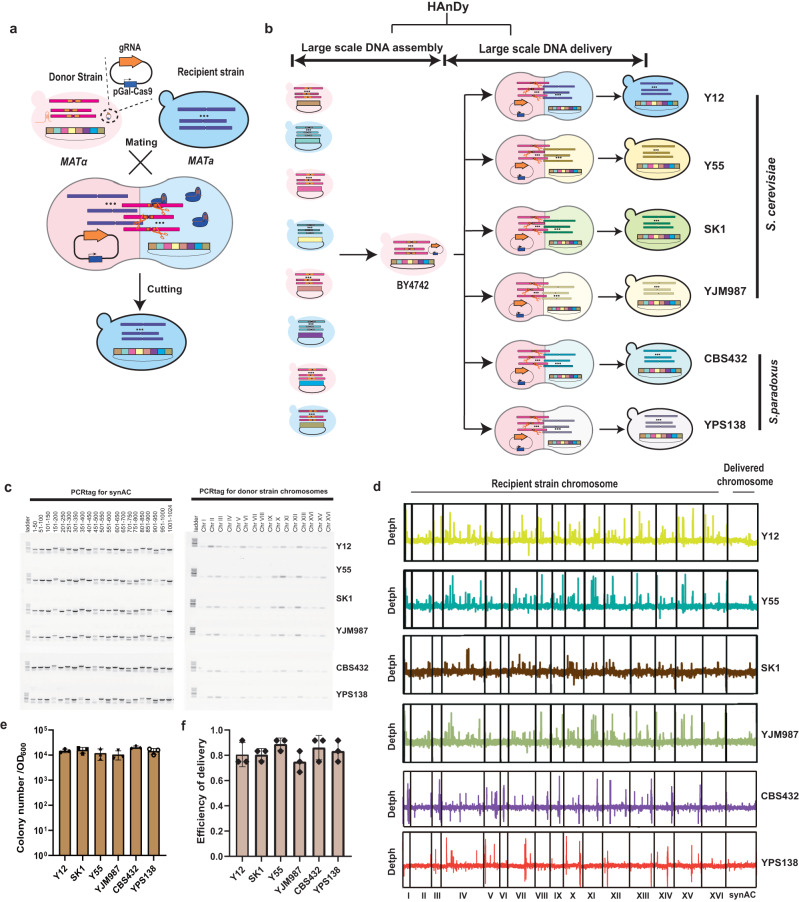
Delivery of the megabase synAC by programmed haploidization. **a** Mechanism for haploidization-based DNA delivery. **b** Process of HAnDy-mediated large-scale DNA assembly and delivery. **c** PCRTag analysis of the delivered synAC and donor strain genome. The 21 PCRTags against the synAC and the 16 PCRTags against the donor strain genomic DNA were used in this trial. **d** Whole-genome sequencing analysis of programmed haploidization-based delivery in six different strains. **e** Number of colonies for delivery of the synAC to six different yeasts. The data were obtained from the screening plate per 10^7^ cells from the induced medium. Data are shown as mean ± SD (*n* = 3 three biological replicates). **f** Efficiency of delivery of the synAC to 6 different yeasts. The efficiency of delivery was characterized by PCR and flow cytometry. A total of 12 strains were tested for each replicate and the error bars indicate the standard deviations of all biological replicates (*n* = 3).

Delivery of large-scale DNA to different hosts is essential for expanding the application of synthetic genomics.^14^ However, the existing inefficient and complex large-scale DNA delivery methods have restricted progress toward this goal.^16^ Here, we established a convenient method for the delivery of the assembled large-scale DNA. Specifically, programmed haploidization is active upon mating of the assembly host with the recipient strain, enabling selective elimination of the assembly host genome and delivery of the assembled DNA to the recipient strain (Fig. 5a). As a pilot study, we delivered the 1.024 Mb assembled synAC from the *S. cerevisiae* BY4741 to four *S. cerevisiae* (Y12, SK1, Y55, and YJM987) strains and two *Saccharomyces paradoxus* (CBS432 and YPS138) strains (Fig. 5b). Firstly, we transferred the synAC from assembled strain BY4741 to BY4742 to switch mating type using genome elimination-mediated haploidization, to facilitate the subsequent delivery through mating (Supplementary information, Fig. S10). Subsequently, we used the BY4742 strain as the donor strain for delivery of the synAC to other strains. The BY4742 strain harboring synAC was mated with different recipient strains in a galactose medium, and the induced Cas9 protein and sgRNA selectively targeted the donor host genome. The PCR results indicated that all 16 PCRTags against the donor genomic DNA were lost, whereas all 21 PCRTags against the synAC were preserved (Fig. 5c). Whole-genome sequencing further verified that the complete synAC was delivered to the six recipients and the entire donor genome was eliminated (Fig. 5d). For delivery of the synAC, we easily obtained ~10^4^ colonies per 10^7^ induced cells (Fig. 5e), and among which ~80% of the recipients acquired the synAC (Fig. 5f). These results indicate that stimulating the haploidization system in the mating process enables selected delivery of the megabase DNA to different unmodified recipient strains.

### Properties of hosts harboring the synAC

**Fig. 6:**
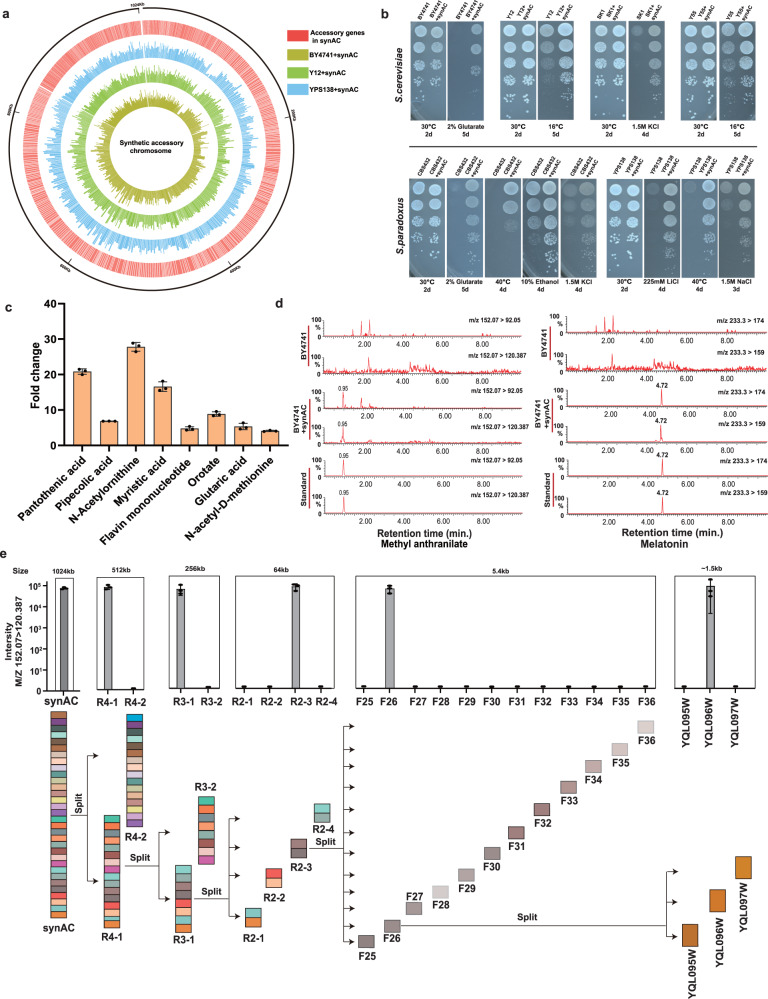
Properties of host harboring the synAC. **a** Transcripts of the synAC in different genetic backgrounds (BY4741, Y12 and YPS138). The expression of each accessory gene is represented as the value of log_2_(FPKM). The FPKM for each gene was taken by the average of three biological replicates. **b** Phenotypes of yeast strains harboring the synAC under various conditions. See Supplementary information, Fig. S8 for a complete view of phenotype testing. **c** Fold change of eight endogenous metabolites in BY4741+synAC. Data are presented as mean ± SD of three biological replicates. **d** Confirmation of melatonin and methyl anthranilate by authentic standards in BY4741+synAC using MRM. MRM traces are shown for metabolite and for authentic standard using two different precursor ion/product ion transitions. Strain BY4741 harboring a plasmid pRS416 was used as control. Chromatogram traces are representative of three biological replicates. **e** A hierarchical narrow-down mapping strategy to decipher the genes related to the biosynthesis of methyl anthranilate in synAC. 23 strains harboring different lengths of the synAC were tested in five rounds of narrow-down test. Data are presented as mean ± SD of the spectral intensity (*n* = 3) for the highest precursor/product ion transition in MRM. For **c**, **d**, **e**, strains were cultured in selective SC-Ura medium with 2% dextrose at 30 °C for 48 h before LC-MS/MS analysis.

To confirm the transcription of synAC in various recipients, we selected three strains BY4741+synAC, Y12+synAC, and YPS138+synAC for analysis by RNA sequencing (RNA-seq). The results show that all 183 non-homologous genes and 359 orthologous genes are transcribed in the three recipients (Fig. 6a). To further investigate the phenotypical effects of synAC in various recipients, we characterized the phenotypes of the strains under various conditions, such as carbon source, temperature, osmotic pressure, heavy metals, etc. Serial dilution assay and growth curves demonstrate that synAC, as a movable functional module, can effectively promote host adaptations under a variety of environmental stresses (Fig. 6b; Supplementary information, Figs. S11, S12). Compared to the wild-type (WT) strains, the *S. cerevisiae* strains Y12+synAC and Y55+synAC grew better at low temperature of 16 °C and the *S. paradoxus* strain CBS432+synAC had obvious growth advantage in medium containing 10% ethanol (Fig. 6b). Surprisingly, the synAC endowed thermotolerance to *S. paradoxus* CBS432 at 40 °C and conferred BY4741 and CBS432 the ability to utilize glutarate (Fig. 6b), which revealed a gain-of-function effect of synAC.

In general, *S. cerevisiae* shows a growth advantage over its distant relative *S. paradoxus* at high temperature, and it is speculated that several *S. cerevisiae* specific genes may contribute to the thermotolerance.^39^ Through synAC, we introduced 532 accessory genes from the collection of 1011* S. cerevisiae* isolates that may confer the thermotolerance to *S. paradoxus* CBS432. Furthermore, we also found that another *S. paradoxus* YPS138+synAC grew better than the WT strain at 40 °C (Fig. 6b). Glutarate is an intermediate of lysine degradation, which can be utilized as a carbon source through glutaryl-CoA dehydrogenation pathway or glutarate hydroxylation pathway.^40^ However, it was reported that *S. cerevisiae* BY4741 is incapable of using glutarate as its sole carbon source.^29^ In our study, we found that BY4741+synAC could grow on medium with glutarate as the sole carbon source, and the key intermediate S-2-hydroxyglutarate from glutarate to the citrate cycle was also identified in the metabolites of BY4741+synAC (Supplementary information, Fig. S13). We speculate that the synAC enables utilization of glutarate by completing the glutarate hydroxylation pathway.

To characterize global effect of synAC on host metabolism, untargeted metabolomics and authentic standard validation were employed to analyze strains harboring the synAC. Through quantitative analysis of metabolites using authentic standards and multiple reaction monitoring (MRM), we confirmed the enrichment of eight endogenous metabolites, including pantothenic acid, myristic acid, and pipecolic acid (Fig. 6c). Interestingly, we discovered 9 potential new compounds in the strain harboring the synAC which shared the same retention time with authentic standards (Supplementary information, Fig. S14).

In addition, we detected an aromatic compound methyl anthranilate (MANT) (Fig. 6d), which has been widely used in flavoring foods.^41^ However, the biosynthetic pathway has only been reported in plants.^42^ To identify novel genes related to the biosynthesis of MANT in synAC, we employed a hierarchical narrow-down mapping strategy (Fig. 6e). Through five rounds of narrow-down tests, we identified a novel gene, *YQL096W*, which is involved in the biosynthesis of MANT. This finding was further confirmed by episomal expression of the single *YQL096W* gene in BY4741 (Fig. 6e). The novel gene *YQL096W*, originated from *S. cerevisiae* YJM1400 (isolated from palm wine fermentation), is predicted to be an S-adenosyl-l-methionine (SAM)-dependent methyltransferase by CLEAN, which may catalyze a reaction of transferring a methyl group to anthranilic acid, similar to the biosynthesis pathway of MANT in plants.^41^ In addition, we noticed another potential new compound melatonin which is also derived from tryptophan (Fig. 6d). Melatonin is a hormone that regulates human sleep. Previous studies have indicated that it is also present in the wine yeasts of ARM, QA23, and EC1118,^43–45^ and these strains have been incorporated into the strain collection of our design of synAC. The accessory genes from these strains may contribute to the production of melatonin in BY4741+synAC. Collectively, these results showed that synAC as an expanded functional module could enhance the host’s adaptation to various conditions and empower biosynthetic capacity to produce new compounds.

## Discussion

We have established an efficient and convenient approach for the assembly and subsequent delivery of large-scale DNA in yeast. Compared with other large-scale DNA assembly methods (Supplementary information, Table S1), HAnDy is an easy-to-use method. The main experimental operation of HAnDy is simply co-culturing yeast cells, avoiding the complex, time-consuming, and inefficient process of purification and introduction of large-scale DNA, which is a general bottleneck for iterative assembly of large-scale DNA. Considering the straightforward principle of this approach and capacity of the yeast centromeric vector,^46,47^ it is reasonable to expect the application of HAnDy in assembly of larger-scale DNA (>10 Mb), which is important for exploration of the upper size limits of yeast assembly and synthetic genomics study of higher organisms. Moreover, HAnDy is also an efficient delivery method that enables transfer of assembled large-scale DNA to various recipient strains by spontaneous mating and programmed haploidization, largely extending the scope of applications of synthetic chromosomes. The underlying principle of this delivery method — fusion of donor cell and recipient cell followed by elimination of the donor genome — could be expanded to other methods of cell fusion,^48–50^ such as fusion of yeast protoplasts and mammalian cells.

Complex traits are often predicted to be governed by multiple genes that function systemically and synergistically.^51,52^ In this study, we designed and assembled a megabase synAC encoding a total of 542 accessory genes from the collection of 1011 *S. cerevisiae* isolates, which is an extrachromosomal functional module harboring a larger number of exogenous genes than previous reports.^53,54^ Introducing large-scale exogenous genetic elements enables the emergence of complex functions, such as various environmental tolerances and the ability to produce new compounds. However, deciphering the genetics of complex traits is challenging due to the underlying complex gene interactions.^55–57^ In this study, a total of 23 strains were constructed and characterized to map a single gene-related trait of MANT biosynthesis. For multiple gene-related traits, the workload of genetic dissection would dramatically increase. For instance, in the validation process of a predicted novel compound scopolamine, we noted that deletion of three regions in the synAC led to incapability of scopolamine production, suggesting that biosynthesis of this compound may involve a multiple gene-related complex pathway (Supplementary information, Fig. S15). In the design of synAC, we introduced numerous site-specific recombination sites upstream of each accessory genes, which enable generation of structural variation libraries with the expression of Cre and Vika recombinases. The structural variation libraries which contain multi-gene random deletions in synAC can facilitate the systematical dissection of relationship between genotypes and complex traits. Our strategy of combinational import of hundreds of genes into a single yeast by design, assembly and delivery of a synAC, provides an extraordinary approach to diversify the host properties and systemically mine the complex traits. In addition, it may also provide a model to investigate the impact of overexpression of a large number of proteins on intracellular resource allocation.^58,59^

## Materials and methods

### The design principle for selecting accessory genes and promoters

We categorized the 1715 accessory genes from the pan-genome of 1011 yeasts^23^ into two groups: orthologous and non-homologous. Among the orthologous genes, we selected 359 genes who share > 80% similarity with S288C through bi-directional Blast hit (BBH) analysis.^29^ For non-homologous genes, we selected 183 genes which are not significantly similar to S288C using the standard Blast web service and present in more than five strains among the 1011 isolates.

According to the types of accessory genes, we also classified the promoters into two categories: endogenous promoters and synthetic promoters. The promoter of orthologous accessory gene was derived from the corresponding gene’s promoter in S288C. To avoid the repetitive use of synthetic promoters, we leveraged the most extensive synthetic promoter library available, encompassing a vast pool of 100 million synthetic promoters.^31^ Specifically, we chose the top 183 promoters based on their transcriptional strength in the library.

### Construction of strains with modified centromeres

The *S. cerevisiae* BY4742 was used as the starting strain, in which a Cas9 uniform target site XT2 and a *URA3* gene were inserted adjacent to the centromeres of all 16 chromosomes. Construction of the 16 chromosome-modified strains was carried out in three steps (Supplementary information, Fig. S3). First, sgRNA arrays targeting chromosomes 1–4 and the corresponding donor fragments were introduced into BY4742. For each chromosome, ~1 μg of the chromosome targeting cassette HR1–4 (containing 400 bp homology arms for each centromere, URA3 cassette, and CRISPR protospacer sequence XT2: GGTGTAACGTAGACTCACAGTGG) and sgRNA array plasmid pRS42H-sgChr1–4 were co-transformed into *S. cerevisiae* BY4742 cells harboring the constitutively expressed Cas9 plasmid (pRS415-Cas9) using a standard lithium acetate transformation protocol.^60^ The transformed cells were plated on the synthetic dropout medium (SC-Ura-Leu+HYG) without uracil and leucine. Positive colonies were verified by PCR and Sanger sequencing. Second, positive colonies were grown in 5 mL of SC-Ura-Leu liquid medium at 30 °C for 24 h, and HR5–8 and sgRNA array plasmid pRS42B-sgChr5–8 were introduced by standard lithium acetate transformation. The transformed cells were plated on the synthetic dropout medium (SC-Ura-Leu+Ble) without uracil and leucine, and the positive colonies were verified by PCR and Sanger sequencing, and so forth. Similarly, BY4741-Chr9–16^XT2-URA^ was constructed. sgRNA array for wtChr1–16 and Cas9 plasmid (pRS415-Cas9) were introduced into BY4742-Chr1–8^XT2-URA^ and BY4741-Chr9–16^XT2-URA^, respectively. Third, after selection for correctly transformed colonies, BY4742-Chr1–8^XT2-URA^ (harboring sgRNA array for wtChr1–16) and BY4741-Chr9–16^XT2-URA^ (harboring pRS415-Cas9) were plated on the synthetic dropout medium (SC-Ura-Leu) at 30 °C, respectively. Each colony of them was selected and added into 5 mL of YPD liquid medium to mate. Then PCR verification was performed to select correct BY4742-Chr1–16^XT2-URA^ colony. Following the above process, XT1 (GCGGGATGGTGTCCCCAGGGCGG) and FCY1 were integrated at the centromeres of the 16 chromosomes in BY4741 to construct BY4741^XT1-FCY^ strain.

### Haploidization by genome elimination in yeast

The CRISPR/Cas9-induced DSBs at all centromeres were triggered by co-culturing haploid strains BY4741 and BY4742^XT2-URA^, which harbor Cas9 (pRS415-Cas9) and gRNA plasmids (pRS42H-sgXT2), respectively. Yeasts with different mating types were incubated in corresponding selective media overnight. One milliliter of yeast culture with different mating types was harvested and washed twice with sterile ddH_2_O. Two hundred microliters of each culture were then added to 5 mL YPD (in which each strain grew well) and incubated at 30 °C with shaking at 220 rpm for 8 h. Cells from 1 mL of the mating solution were subsequently harvested and washed with sterile ddH_2_O twice. Ten microliters of the solution and 200 µL of sterile ddH_2_O were mixed together, plated onto selective medium (SC-Leu+Hyg+5-FOA), and then incubated at 30 °C for 2–3 days.

### PCRTag analysis of haploidized strain

To verify the elimination of BY4741^XT1-FCY1^ and BY4742^XT2-URA3^ chromosomes, we used the junctions between *FCY1* or *URA3* and chromosomes at the BY4741^XT1-FCY1^ or BY4742^XT2-URA3^ centromere as specific PCRTags by colony PCR to distinguish the chromosome-modified and WT strains. Briefly, a single yeast colony was resuspended in 10–40 µL of 20 mM NaOH and placed in the thermocycler. Yeast colony suspensions were boiled at 95 °C for 5 min and then cooled at 4 °C for at least 5 min before PCR was performed. 1 µL of yeast lysate was used as a template in a 10 µL 2× Rapid Taq Master Mix (Vazyme P222-AA) with 0.25 µM of primers. PCR program: 95 °C, 5 min; 30× (95 °C, 20 s; 55 °C, 90 s; 72 °C, 1 min); 72 °C, 5 min; 4 °C, hold. PCR products were separated on a 1% agarose gel containing ethidium bromide. 2 kb Plus II DNA Ladder (TransGen BM121-01) was used as a molecular weight standard.

### DNA content measurement by flow cytometry

First, a single yeast colony was picked and inoculated into 5 mL of YPD or SC medium and cultured until reaching the logarithmic phase. A volume of 3 mL of the culture was centrifuged at 5000 rpm for 2 min to collect the cells, which were washed twice with ddH_2_O. The cell pellet was resuspended in 1 mL of ddH_2_O, and the optical density at 600 nm (OD_600_) of the cell suspension was measured. The haploid and diploid cell suspensions were diluted with ddH_2_O to OD_600_ of 1 (~10^7^ cells/mL). For fixation, 10^7^ cells were treated with 70% ethanol at room temperature for 1 h. The cells were then centrifuged at 5000 rpm for 2 min and the cell pellets were resuspended in 50 mM sodium citrate buffer. After centrifugation, the cell pellets were collected and resuspended in 975 μL of sodium citrate buffer, and 25 μL of RNase A (10 mg/mL) was added, followed by incubation at 50 °C for 1 h. Subsequently, 50 μL of proteinase K (20 mg/mL) was added and incubated at 50 °C for 1 h. A 1:10 dilution of SYBR dye in Tris-EDTA buffer (pH 8.0) was prepared, and 20 μL of the diluted SYBR dye was added to the above system for dark staining at room temperature for 1 h. Triton X-100 was added to a final concentration of 0.25%, and the sample was vortexed. The cell suspension was transferred to a 12 mm × 75 mm tube, and the cell size and total DNA content were measured using a flow cytometer to determine the ploidy of the cells. The distributions of FITC-A were processed to identify the two main density peaks corresponding to the cell populations in G1 and G2 phases. Flow cytometry was performed using a BD Aria III. The data were analyzed with FlowJo (Treestar).

### Evaluation of the mating ability using *Tester* a and *Tester* α

Two different mating type strains lacking *HIS1*, referred to as *Tester* a and *Tester* α, were used. Both strains were selected and cultured overnight in 5 mL YPD at 30 °C with shaking at 220 rpm. Two hundred microliters of both culture solutions were plated onto SD solid medium and recorded as test plates. After haploidization, the strains were copied onto test plates, respectively. The SD solid medium plates were cultured at 30 °C for 24 h.

### Construction of the iterative assembly vectors

Iterative assembly parts were constructed in this study to facilitate the genetic manipulation. Four distinct target sites for the gRNA–Cas9 complex, each consisting of a 23 bp sequence containing a protospacer adjacent motif, were designed for iterative assembly. The gRNA expression cassettes, namely gRNA-S1, gRNA-S2, gRNA-S3, gRNA-S4, and gRNA-S5, corresponding to the five target DNA sites (S1 site: cggtggacttcggctacgtaggg, S2 site: gctgttcgtgtgcgcgtcctggg, S3 site: acttgaagattctttagtgtagg, S4 site: cgccgctccgagggccgcacggg, and S5 site: gttgcaaatgctccgtcgacggg), were generated using PCR and overlap-PCR techniques. The selective marker genes *HIS3* (located on the plasmid backbone), *LEU2* (located on the plasmid backbone), *URA3*, and *LYS2*, were amplified from plasmids pRS413, pRS415, pRS416, and BY4742 genome, respectively. The homologous arm fragment HR (~400 bp) was amplified using PCC1 plasmid as the template, which was similar to the plasmid homologous arm sequence.

### Construction of initial fragments using TAR assembly in *S. cerevisiae*

To facilitate the future assembly of the synAC, sets of neighboring fragments consisting of five or six fragments were introduced into *S. cerevisiae*. These fragments were used to construct 32 initial fragments (~32 kb) using TAR assembly. To construct the initial fragment plasmids, functional vectors containing iterative assembly parts and homologous arms (500 bp homologous arms designed to be added to the ends of DNA fragments) were pre-constructed. The NEBuilder HiFi DNA Assembly Master Mix from NEB was employed to assemble the vectors and iterative parts into the pre-constructed functional vectors. Subsequently, the pre-constructed vectors were amplified using the KOD-one (a kind of DNA polymerase) PCR Master Mix from TOYOBO. Five or six linear fragments (100–200 ng of each fragment) and the pre-constructed functional vector (~100 ng) were co-transformed into BY4741^XT1-FCY^ and BY4742^XT2-URA^ yeast strains according to the design outlined in Supplementary information, Data S2.

### DNA assembly via HAnDy

Single colonies were inoculated into 5 mL of SC medium and grew overnight at 30 °C with shaking at 250 rpm until the OD_600_ reached a range of 4–5. Approximately equal amounts of two neighboring haploid cells (~200 μL) with opposite mating types were co-cultured in 5 mL of fresh YPD medium. Mating occurs when haploid cells with opposite mating types are co-cultured. The mating process involves spontaneous assembly of DNA fragments and programmed haploidization facilitated by the orthogonal-cut CRISPR/Cas9 system in diploid cells. After co-culturing for 8–12 h, a volume of 0.5 mL of the culture was centrifuged at 5000 rpm for 2 min to collect the cells, which were washed twice with ddH_2_O. The cell pellet was resuspended with ddH_2_O and diluted to an OD_600_ of 1 (~10^7^ cells/mL), and 20 μL of the diluted solution was plated on a selective medium. The plates were then incubated at 30 °C for 1–2 days. Colonies that grew on the selective medium were picked and verified for successful assembly through PCR analysis of the newly formed junctions and chromosome elimination. The colony PCR validation was performed as described previously. For each assembly, PCR verification was carried out on the junctions at both ends of each fragment and haploidized yeast. Positive colonies were picked to validate the DNA content by flow cytometry further. The positive haploid colonies confirmed by PCR sequencing were inoculated and grown in 3 mL of SC liquid medium until saturation at 30 °C to eliminate the plasmid harboring the haploidization system. After 24 h, 1 µL of the culture was plated and grown on SC selective medium.

### PFGE

The PFGE protocol was modified,^61^ and a single colony was inoculated into 5 mL of YPD overnight with shaking at 30 °C. One milliliter of the overnight culture was transferred to a tube and centrifuged at 1200× *g* for 2 min at room temperature. Cells were washed twice in solution I (0.05 M EDTA, 0.01 M Tris, pH 7.5) and resuspended in 150 µL of solution I with 10 µL of zymolyase (2 mg/mL zymolyase 20 T, 10 mM sodium phosphate, pH 7.5). Cells were placed in a 42 °C heat block. Two hundred and fifty microliters of agarose solution (1% (w/v) low-melting temperature agarose, 0.125 M EDTA, pH 7.5) was preincubated at 42 °C and mixed with cells by pipetting with a wide-bore pipette tip in the tube. The tube was placed on ice immediately, and 400 µL of LET (0.5 M EDTA, 0.01 M Tris, pH 7.5) was added. The tube was incubated for 8–10 h overnight at 37 °C and placed on ice for 10–20 min, and then the agarose plug in the tube was transferred to a 15 mL Falcon tube. Four hundred microliters of NDS (0.5 M EDTA, 0.01 M Tris, pH 7.5, 1% (w/v) sodium lauryl sarcosine, 2 mg/mL proteinase K) was added and incubated overnight at 50 °C. The tubes were placed on ice for 10 min. The NDS was exchanged with solution I and rocked/swirled gently at room temperature for 1 h, and the wash was repeated three more times. Plugs were stored in fresh solution I at 4 °C. The electrophoresis was performed in one stage, and the gel was prepared with 1% low-melting agarose and 1× Tris/borate/EDTA (TBE) buffer. The electrophoresis conditions were as follows: switch time of 60–120 s, run time of 20 h, angle of 120°, and voltage of 6 V/cm. For the second time, the agarose plugs were removed from the agarose gel and digested for 2–3 h by a restriction enzyme (NEB) before the second PFGE analysis. The conditions of the PFGE program were set as follows: the voltage was 6 V/cm at an angle of 120°, the switch time ranged from initial values of 60 s to a final value of 120 s, the temperature was 10 °C, and the total time was 22 h. Using WT BY4742 genome agarose plugs as a marker, the sizes of the assembled large DNA constructs (200 kb–1.5 Mb) could be validated.

### Whole-genome sequencing and RNA-seq

RNA-seq workflow is as follows: yeast cells harboring synAC and the control strain harboring pRS416 were cultured overnight in 3 mL of SC-Ura medium at 30 °C. The cultures were added to 10 mL of fresh SC-Ura medium and incubated until the OD_600_ reached ~0.8. Three parallel samples were set for each yeast strain harboring the synAC. The RNA extraction was conducted according to the standard procedure. The samples were tested using the BGISEQ-500 platform, and each sample produced an average of 6 GB of data. The average alignment rate of the sample against the reference genome was 95.79%. The sequencing data is called raw reads or raw data, and then quality control (QC) of the raw reads is performed to determine whether the sequencing data is suitable for subsequent analysis. The filtered sequencing clean data were aligned to the reference genome using the Hisat2 (v2.0.1) software for short-read alignment, with default parameters.^62^ To distinguish between the expression of endogenous and accessory genes, we employed a sequence alignment approach to select specific 30-bp tags within each accessory gene for specific differentiation of endogenous and accessory genes, following the previous study.^63^ For whole-genome sequencing, the strain samples were prepared and analyzed according to the standard protocols.^19^

### DNA delivery via HAnDy

Single colonies of assembly hosts and recipient strains were inoculated into 5 mL of SC medium and grew overnight at 30 °C with shaking at 250 rpm until the OD_600_ reached 4–5. A volume of 0.3 mL of the culture was centrifuged at 5000 rpm for 2 min to collect the cells, which were washed three times with 1 mL ddH_2_O. Approximately equal amounts of haploids with opposite mating types were co-cultured in 5 mL of fresh YPD medium containing 2% galactose and 3% raffinose instead of 2% glucose. The initial OD_600_ of the co-culture was set to 0.3. After co-culturing for ~12 h, 2 mL of the culture was centrifuged at 5000 rpm for 2 min to collect the cells, which were washed twice with ddH_2_O. The cell pellet was resuspended with ddH_2_O and diluted to an OD_600_ of 1 (~10^7^ cells /mL), and 20 μL of the diluted solution was plated on a selective medium containing 1 mg/mL 5-FOA. The plates were then incubated at 30 °C for 1–2 days. Colonies that grew on the selective medium were screened and picked for verification. Colony PCR validation was performed as described before. Positive colonies were picked to further validate the DNA content by flow cytometry and whole-genome sequencing.

### Serial dilution assays on various types of medium

Yeasts were incubated in 5 mL of liquid SC medium (2% glucose, 0.2% dropout mixture, 6.72 g/L yeast nitrogen base) overnight at 30 °C with rotation at 220 rpm, after which 200 µL of the culture was transferred to 5 mL of SC medium at 30 °C with rotation at 220 rpm and then grown to an OD_600_ of 0.5. The cultures were serially diluted in 10-fold increments in ddH_2_O and, and ~5 µL of the diluted solution was spotted from the lowest to the highest concentrations on the corresponding selective solid SC medium. These plates were incubated for 3–5 days at 30 °C or other temperature. For the sole carbon source culture, glucose (2%) was replaced by corresponding carbon source (2%). For the osmotic pressure and heavy metal conditions, the SC medium was supplemented with 1.5 M NaCl, 1.5 M KCl, 225 mM LiCl and 10% ethanol, and 0.2 mM Cd(NO_3_)_2_, respectively. For the temperature tolerance conditions, routine SC medium was used.

### Untargeted metabolomic sample preparation and extraction

The preparation and extraction of metabolomic samples were performed following standard protocols modified from.^51^ Briefly, the yeast strain was cultured in 5 mL of SC medium and incubated with shaking at 30 °C for 24 h. Subsequently, 5 mL of culture sample was collected and centrifuged at 1200 × *g* for 5 min at 4 °C. The cell pellet was washed twice with Milli-Q water and immediately submerged in a prechilled solution of 60% (v/v) methanol/water to quench the reaction rapidly. After a 30-s incubation at 40 °C, the samples were centrifuged at 4000 × *g* for 5 min at 4 °C to collect the cell pellets. The cells were then washed twice with phosphate-buffered saline at 4 °C, followed by a final wash with Milli-Q water to remove any residual culture medium. The cell pellets were collected by centrifugation at 4000 × *g* for 5 min at 4 °C. To extract the metabolites, 700 μL of an extraction solvent containing an internal standard (methanol:acetonitrile:water = 4:2:1, v/v/v) was added to the cell pellets. The mixture was vigorously shaken for 1 min and placed in a –20 °C freezer for 2 h. Subsequently, the samples were centrifuged at 25,000 × *g* and 4 °C for 15 min. The supernatant (600 μL) was carefully transferred to a new EP tube, followed by freeze-drying. The dried samples were then reconstituted in 180 μL of a methanol:water solution (1:1, v/v) and vortexed for 10 min until complete dissolution. The reconstituted samples were centrifuged at 25,000 × *g* and 4 °C for 15 min. The supernatant was transferred to a new EP tube and stored at –80 °C until further analysis.

### UPLC-MS/MS analysis

For this experiment, we used a Waters UPLC I-Class Plus (Waters, USA) random Q Exactive high-resolution mass spectrometer (Thermo Fisher Scientific, USA) to separate and detect metabolites. Chromatographic separation was performed on a Waters ACQUITY UPLC BEH C18 column (1.7 μm, 2.1 mm × 100 mm, Waters, USA), and the column temperature was maintained at 45 °C. The mobile phase consisted of 0.1% formic acid (A) and acetonitrile (B) in positive mode and 10 mM ammonium formate (A) and acetonitrile (B) in negative mode. The gradient conditions were as follows: 0–1 min, 2% B; 1–9 min, 2%–98% B; 9–12 min, 98% B; 12–12.1 min, 98%–2% B; and 12.1–15 min, 2% B. The flow rate was 0.35 mL/min, and the injection volume was 5 μL. The mass spectrometry (MS) conditions were as follows: Q Exactive (Thermo Fisher Scientific, USA) was used to perform primary and secondary MS data acquisition. The full scan range was 70–1050 *m/z* with a resolution of 70,000, and the automatic gain control (AGC) target for MS acquisitions was set to 3,000,000 with a maximum ion injection time of 100 ms. The top 3 precursors were selected for subsequent MS/MS fragmentation with a maximum ion injection time of 50 ms and resolution of 17,500, and the AGC was 100,000. The stepped normalized collision energy was set to 20 eV, 40 eV and 60 eV. The electrospray ionization (ESI) parameters were set as follows: the sheath gas flow rate was 40, the Aux gas flow rate was 10, the positive-ion mode spray voltage (|KV|) was 3.80, the negative-ion mode spray voltage (|KV|) was 3.20, the capillary temperature was 320 °C, and the Aux gas heater temperature was 350 °C.

### Metabolite ion peak extraction and metabolite identification

After importing the off-line MS data into compound discoverer 3.2 (Thermo Fisher Scientific, USA) software and analyzing the MS data in combination with the bmdb (BGI metabolome database), mzcloud database, and ChemSpider online database, a data matrix containing information such as metabolite peak area and identification results was obtained. Then, the matrix was further analyzed and processed.

Software: Compound Discoverer v.3.2

Parameter: Parent ion mass deviation: <5 ppm

Mass deviation of fragment ions: <10 ppm

Retention time deviation: <0.2 min.

### Untargeted LC-MS/MS data processing and analysis

#### Data preprocessing

The resulting file was input from Compound Discoverer to MetaX for data preprocessing and further analysis. Data preprocessing included the following: (1) normalizing the data to obtain relative peak areas by probabilistic quotient normalization (PQN);^64^ (2) QC-based robust LOESS signal correction to correct the batch effect;^65^ (3) removing metabolites with a coefficient of variation >30% in their relative peak area in QC samples. PQN is a sample normalization method that can improve comparability between samples via the following steps: (1) obtain an overall reference vector by analyzing the ion intensity distribution in each sample; (2) analyze the correction coefficient between the actual sample and the reference vector for actual sample correction.

QC-RLSC is an effective data correction method in metabolomics, and the method is able to correct experimental sample signals by local polynomial regression fitting signal correction based only on the QC sample.

#### QC

Principal component analysis (PCA) is an unsupervised pattern recognition method for the statistical analysis of multidimensional data. Through orthogonal transformation, a group of variables that may be correlated are converted into a group of linear unrelated variables, which are called principal components after the transformation. This method is used to study how a few principal components can reveal the internal structure between multiple variables while keeping the original variable information. Log transformation and Pareto scaling were mainly used to compute principal components. The PCA plot reflects the real distribution of samples and is mainly used to observe the separation trend between sample groups and whether there are abnormal samples, as well as to reflect the variability between groups and within groups from the original data. For QC samples, the better the QC sample aggregate, the more stable the instrument is, and the better the reproducibility of the collected data.

#### Metabolite functional annotation

Taxonomic and functional annotation of the identified metabolites is a good way to understand the properties of different metabolites. The Human Metabolome Database contains chemical, molecular biology/biochemical and clinical information on metabolites and supports metabolic pathway searches and spectral searches. KEGG pathways form the core of the KEGG database. Numerous metabolic pathways and the relationships among them can be found in this database. In organisms, different metabolites act together to exert their biological functions. The functional annotation of pathways was performed through the KEGG pathway database to determine the main biochemical metabolic pathways associated with the metabolites.

#### Screening the differences between groups

Partial least squares-discriminant analysis (PLS-DA) is a supervised statistical method. It can reflect the differences between classification groups better. This method uses partial least squares regression to establish a model between metabolite expression and sample categories to predict sample categories. Additionally, variable importance in projection (VIP) was used to measure the impact strength and explanatory power of each metabolite expression pattern for the classification and discrimination of each group of samples, helping screen metabolic biomarkers. Generally, a VIP value > 1 could indicate that metabolites have a significant effect on distinct sample categories. After log_2_ transformation of the data, a PLS-DA model was established between the comparative analysis groups (two groups of samples), the scaling method was Pareto, and a 7-fold cross validation was used to validate when building the model. OPLSDA is a combination of OSC and PLS-DA. It is an extension of PLS-DA, which can decompose X matrix information into two types of information related to Y and unrelated to Y, remove information irrelevant to classification, and effectively reduce the complexity of the model without reducing the predictive ability of the model, thereby enhancing the explanatory power of the model.

### MRM analysis of metabolite production

Yeast cultures were pelleted by centrifugation at 3500× *g* for 5 min at 12 °C, and 150 μL aliquots of supernatant were removed for analysis. Metabolites were analyzed by LC-M/MS using a Waters Acquity UPLC and Waters Xevo TQ-XS with the mass spectrometer. Chromatography was performed using an ACQUITY UPLC BEHC18 column (2.1 mm × 100 mm, 1.7 μm; Waters) with 0.1% (v/v) formic acid in water as mobile phase solvent A and 0.1% (v/v) formic acid in acetonitrile as solvent B. The column was operated with a constant flow rate of 0.3 mL/min at 30 °C and a sample injection volume of 5 μL. Chromatographic separation was performed using the following gradient: 0.00–0.5 min, 5% B; 0.50–3.00 min, 5%–24% B; 3.00–4.50 min, 24%–95% B; 4.50–7.00 min, 95% B; 7.01–10.00 min, 5% B. The LC eluent was directed to the MS from 0.01–10.00 min operating with ESI in positive mode, Desolvation temperature of 500 °C, gas flow rate of 11 L/min, and nebulizer pressure of 40 psi.

## Supplementary information

Supplementary information, Fig. S1

Supplementary information, Fig. S2

Supplementary information, Fig. S3

Supplementary information, Fig. S4

Supplementary information, Fig. S5

Supplementary information, Fig. S6

Supplementary information, Fig. S7

Supplementary information, Fig. S8

Supplementary information, Fig. S9

Supplementary information, Fig. S10

Supplementary information, Fig. S11

Supplementary information, Fig. S12

Supplementary information, Fig. S13

Supplementary information, Fig. S14

Supplementary information, Fig. S15

Supplementary information, Table S1

Supplementary information, Data S1–S8

## Data Availability

The whole-genome sequencing and RNA-seq reads are available on NCBI BioProject (PRJNA975219). Raw MS data are available on the MetaboLights (https://www.ebi.ac.uk/metabolights/) under studies of MTBLS7924. Any other relevant data are available from the authors upon reasonable request.
