# Supplementary material for: Convenient synthesis and delivery of a megabase-scale designer accessory chromosome empower biosynthetic capacity
Source: Cell Res. 2024 Feb 8;34(4):309–22. doi: 10.1038/s41422-024-00934-3 (PMC10978979; doi:10.1038/s41422-024-00934-3)
Supplement: Supplementary file 1 — Supplementary information, Fig. S1 [file 41422_2024_934_MOESM1_ESM.pdf]

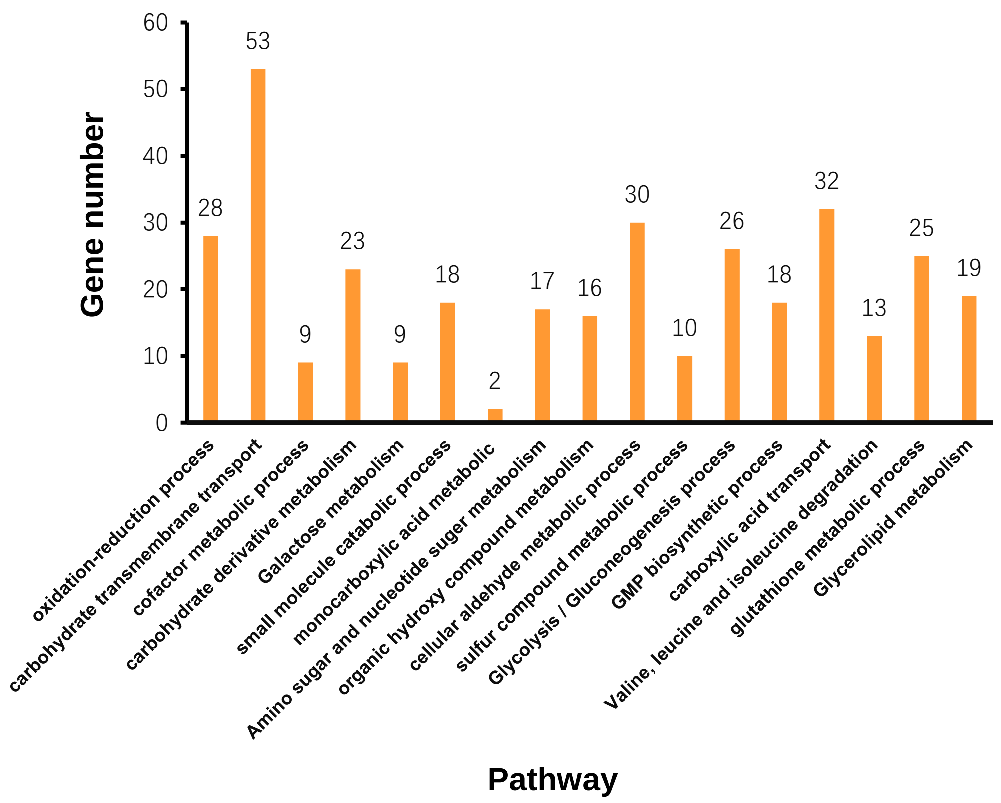

Fig. S1. Clustering of functionally related genes. The 359 orthologous genes can be clustered into 17 clusters by Metascape.
