# Supplementary material for: Convenient synthesis and delivery of a megabase-scale designer accessory chromosome empower biosynthetic capacity
Source: Cell Res. 2024 Feb 8;34(4):309–22. doi: 10.1038/s41422-024-00934-3 (PMC10978979; doi:10.1038/s41422-024-00934-3)
Supplement: Supplementary file 2 — Supplementary information, Fig. S2 [file 41422_2024_934_MOESM2_ESM.pdf]

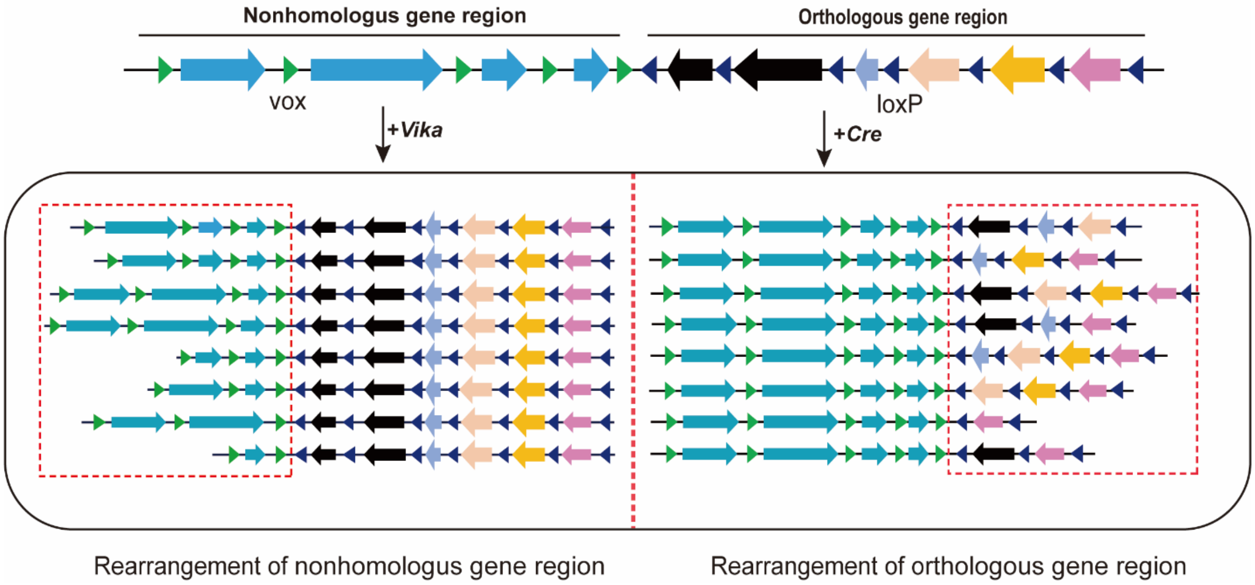

Fig. S2. Cre/loxP and Vika/vox mediated orthogonal rearrangement system. The Cre/loxP (right) and Vika/vox (left) systems are utilized as orthogonal random rearrangement system. Upon induction of Cre recombinase expression, it selectively rearranges the orthologous gene region. Orthogonally, upon induction of Vika recombinase expression, it exclusively rearranges the nonhomologus gene region.
