# Supplementary material for: Convenient synthesis and delivery of a megabase-scale designer accessory chromosome empower biosynthetic capacity
Source: Cell Res. 2024 Feb 8;34(4):309–22. doi: 10.1038/s41422-024-00934-3 (PMC10978979; doi:10.1038/s41422-024-00934-3)
Supplement: Supplementary file 3 — Supplementary information, Fig. S3 [file 41422_2024_934_MOESM3_ESM.pdf]

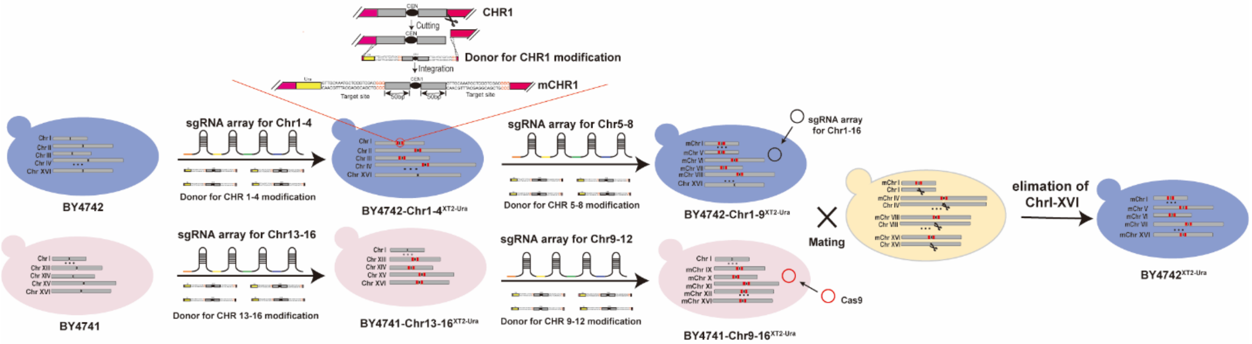

Fig. S3. Construction of strains with modified centromeres. The strains with modifications on all 16 chromosomes was constructed in three steps as described in materials and methods.
