# Supplementary figures and images for: Convenient synthesis and delivery of a megabase-scale designer accessory chromosome empower biosynthetic capacity
Source: Cell Res. 2024 Feb 8;34(4):309–22. doi: 10.1038/s41422-024-00934-3 (PMC10978979; doi:10.1038/s41422-024-00934-3)

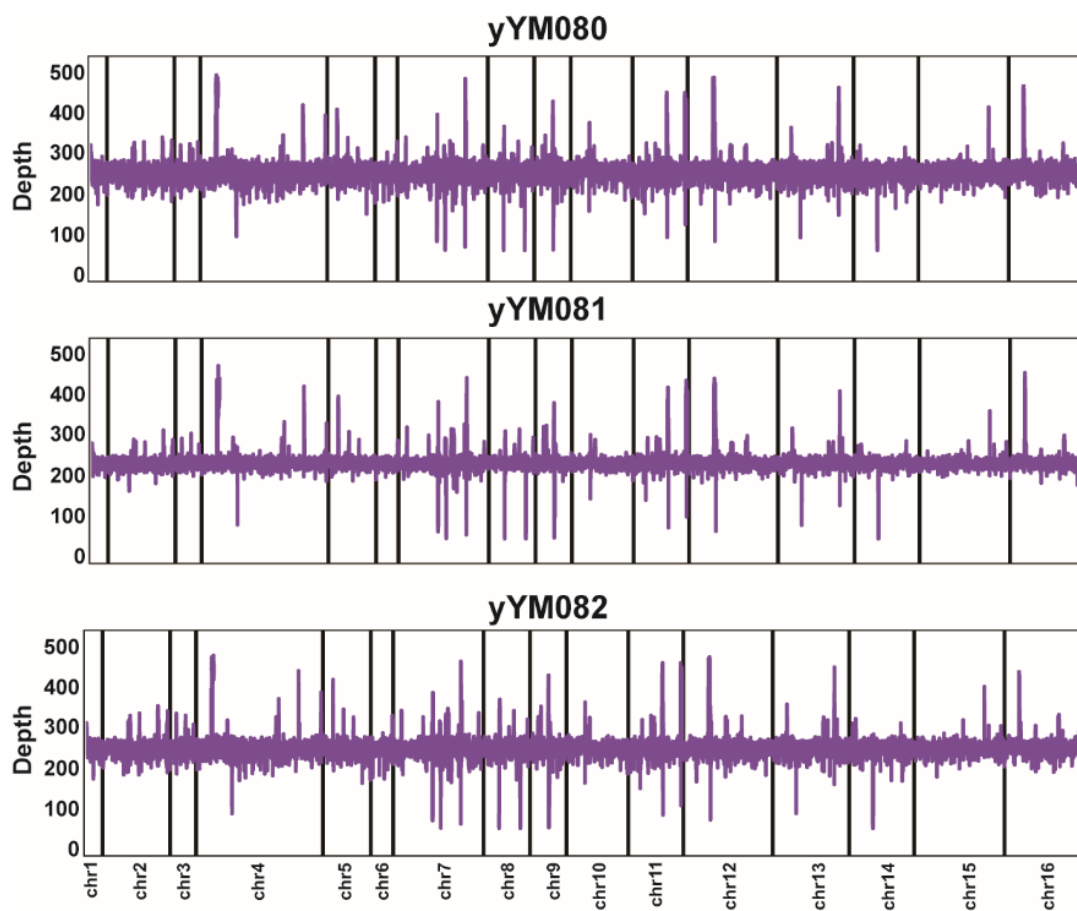

**Fig. S4. The sequencing data of strains after haploidization.**

Supplement: Supplementary file 4 — Supplementary information, Fig. S4 [file 41422_2024_934_MOESM4_ESM.pdf]

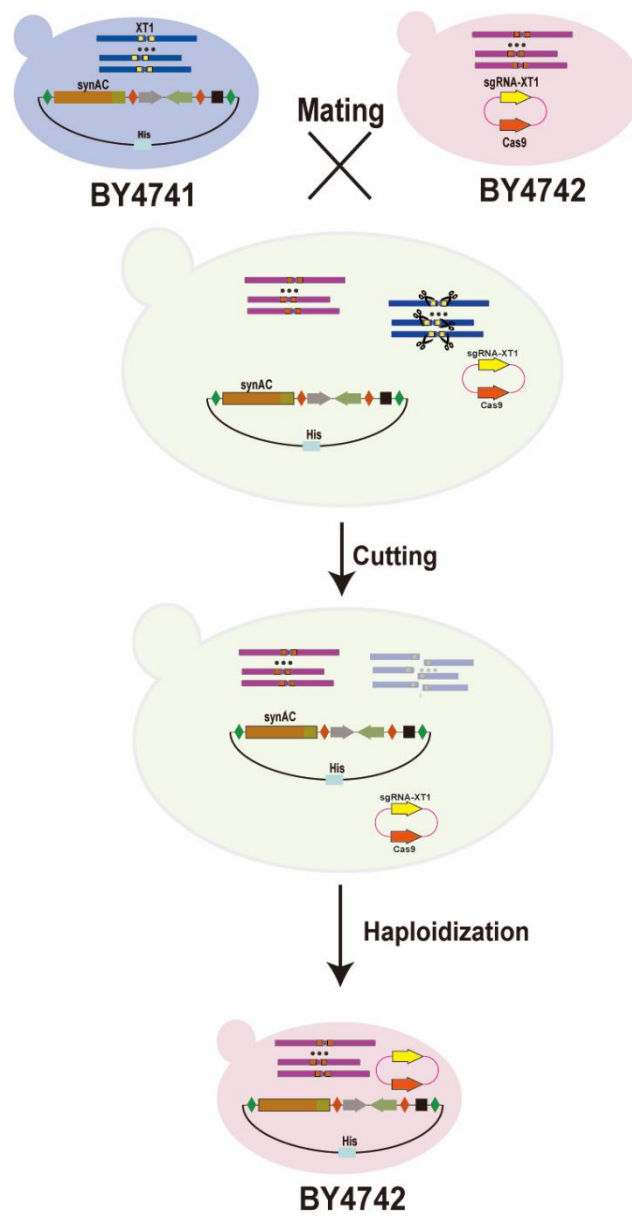

**Fig. S10. Transferred the *synAC* from the assembled BY4741 strain to BY4742 strain.**

Supplement: Supplementary file 10 — Supplementary information, Fig. S10 [file 41422_2024_934_MOESM10_ESM.pdf]
