# Supplementary material for: Convenient synthesis and delivery of a megabase-scale designer accessory chromosome empower biosynthetic capacity
Source: Cell Res. 2024 Feb 8;34(4):309–22. doi: 10.1038/s41422-024-00934-3 (PMC10978979; doi:10.1038/s41422-024-00934-3)
Supplement: Supplementary file 5 — Supplementary information, Fig. S5 [file 41422_2024_934_MOESM5_ESM.pdf]

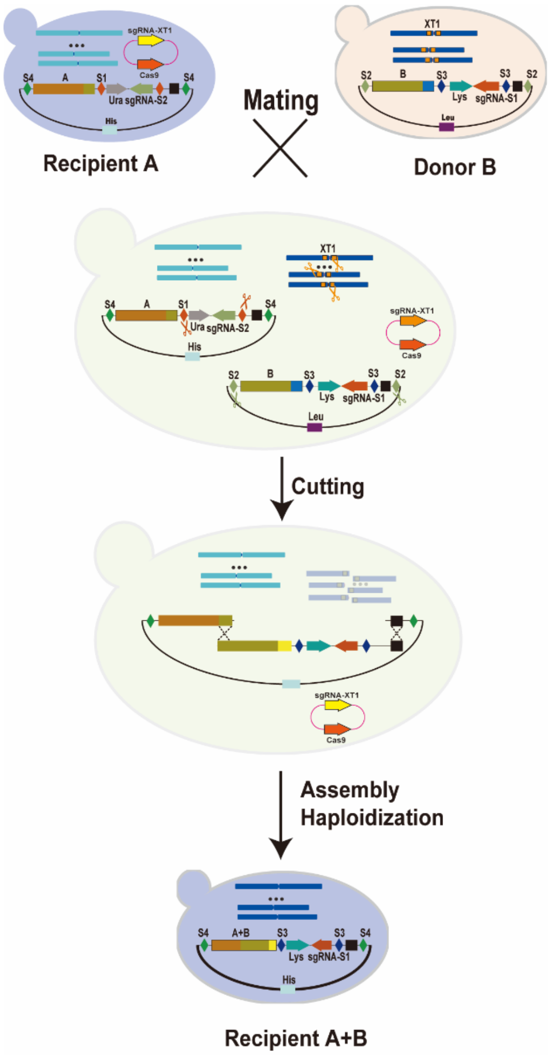

Fig. S5. Detailed schematic illustration for haploidization-based DNA assembly. Blue and red arrows indicate the selection marker and sgRNA expression cassette, respectively. CRISPR protospacer sequences are blue and red rhombuses, respectively. Scissors indicate spacer RNAs that program cleavage within color-matched protospacers. Implementing the iterative assembly process depends on orthologous sgRNAs from the recipient and donor plasmids. Once BY4741 (MATa) is mated with BY4742$^{XT2\text{-}Ura}$ (MATα), sgRNA expressed on the donor plasmid specifically creates DSBs at the recipient selection marker to linearize the receptor plasmids. The sgRNA from the donor plasmid specifically releases the donor fragment and HR sequence. Subsequently, the donor fragment assembled into the recipient plasmid. In this process, the

haploidization plasmid (expressing a specific sgRNA for target XT2) harbored in the recipient strain selectively eliminates the donor genome ($BY4742^{XT2\text{-}Ura}$) to achieve haploidization. The assembled strains are immediately mated with an opposite mating type donor strain to initiate the second round of assembly.
