# Supplementary material for: Convenient synthesis and delivery of a megabase-scale designer accessory chromosome empower biosynthetic capacity
Source: Cell Res. 2024 Feb 8;34(4):309–22. doi: 10.1038/s41422-024-00934-3 (PMC10978979; doi:10.1038/s41422-024-00934-3)
Supplement: Supplementary file 6 — Supplementary information, Fig. S6 [file 41422_2024_934_MOESM6_ESM.pdf]

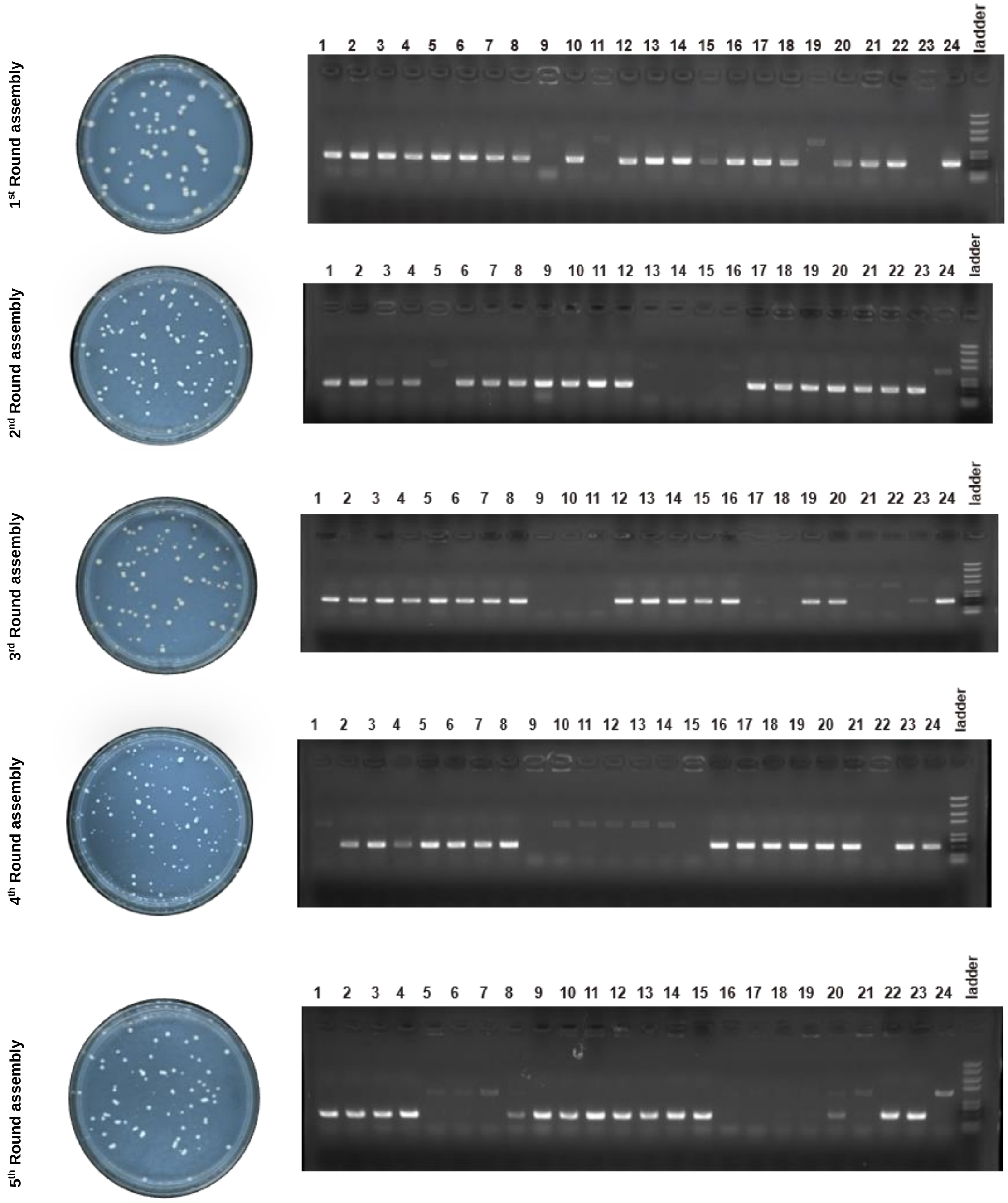

Fig. S6. The colony number and PCR analysis of the assembly junction. Left, the number of colonies obtained on the screening plate per 10^7 cells from the mating medium in each round assembly (dilute 50 times to plate). Right, The PCR result for randomly selected 24 clones on the

screening plate in each assembly round. The primers used for these PCR assemblies are listed in Supplementary Data S6.
