# Supplementary material for: Convenient synthesis and delivery of a megabase-scale designer accessory chromosome empower biosynthetic capacity
Source: Cell Res. 2024 Feb 8;34(4):309–22. doi: 10.1038/s41422-024-00934-3 (PMC10978979; doi:10.1038/s41422-024-00934-3)
Supplement: Supplementary file 7 — Supplementary information, Fig. S7 [file 41422_2024_934_MOESM7_ESM.pdf]

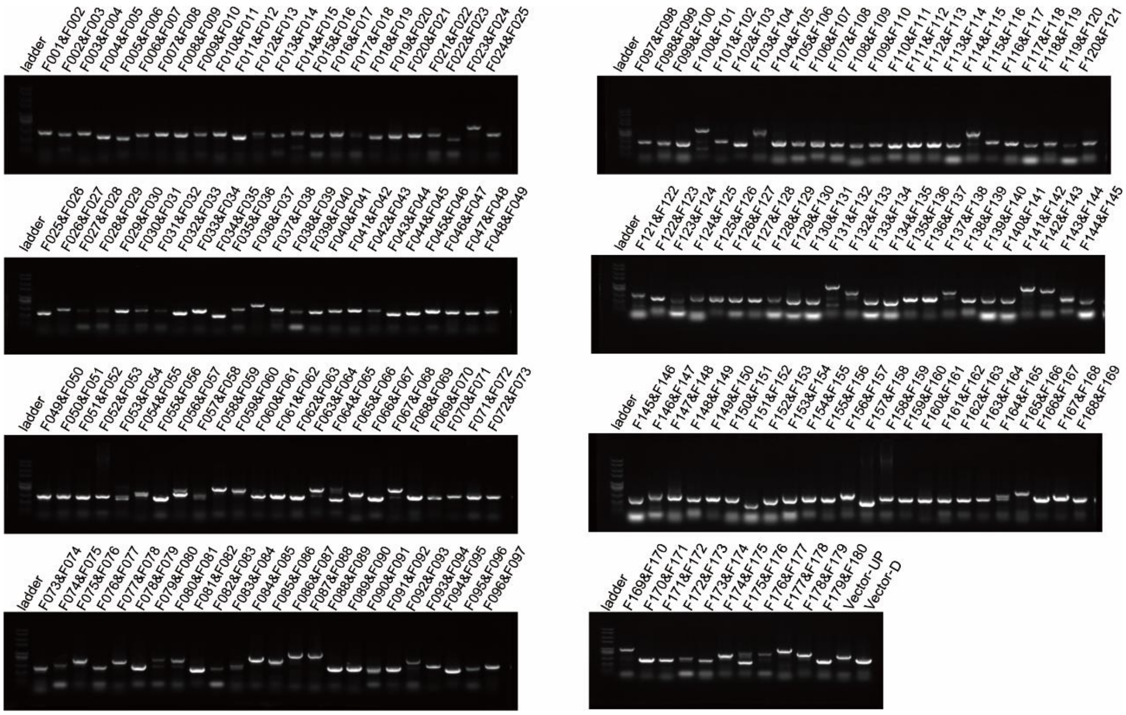

Fig. S7. Complete PCRtag analysis of synAC. Agarose gel showing the strain yYM144 carrying the full 1,024kb synAC verified manually for the presence of all 181 assembly junctions. The primers used for these PCR assemblies are listed in Supplementary Data 6.
