# Supplementary material for: Convenient synthesis and delivery of a megabase-scale designer accessory chromosome empower biosynthetic capacity
Source: Cell Res. 2024 Feb 8;34(4):309–22. doi: 10.1038/s41422-024-00934-3 (PMC10978979; doi:10.1038/s41422-024-00934-3)
Supplement: Supplementary file 8 — Supplementary information, Fig. S8 [file 41422_2024_934_MOESM8_ESM.pdf]

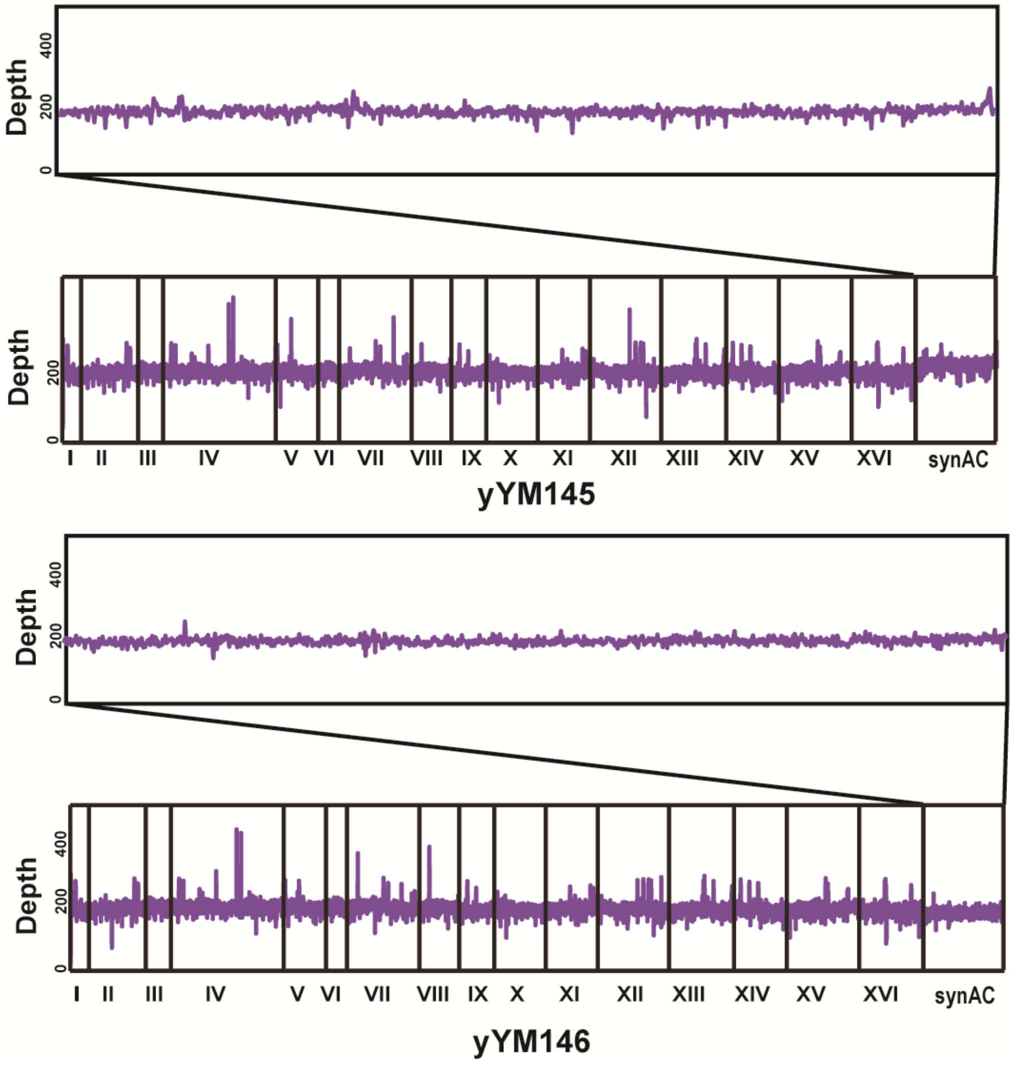

Fig. S8. The sequencing reads depths of all sixteen chromosomes and the synthetic accessory chromosome (synAC) in the final assembly strain yYM145 and yYM146.
