# Supplementary material for: Convenient synthesis and delivery of a megabase-scale designer accessory chromosome empower biosynthetic capacity
Source: Cell Res. 2024 Feb 8;34(4):309–22. doi: 10.1038/s41422-024-00934-3 (PMC10978979; doi:10.1038/s41422-024-00934-3)
Supplement: Supplementary file 9 — Supplementary information, Fig. S9 [file 41422_2024_934_MOESM9_ESM.pdf]

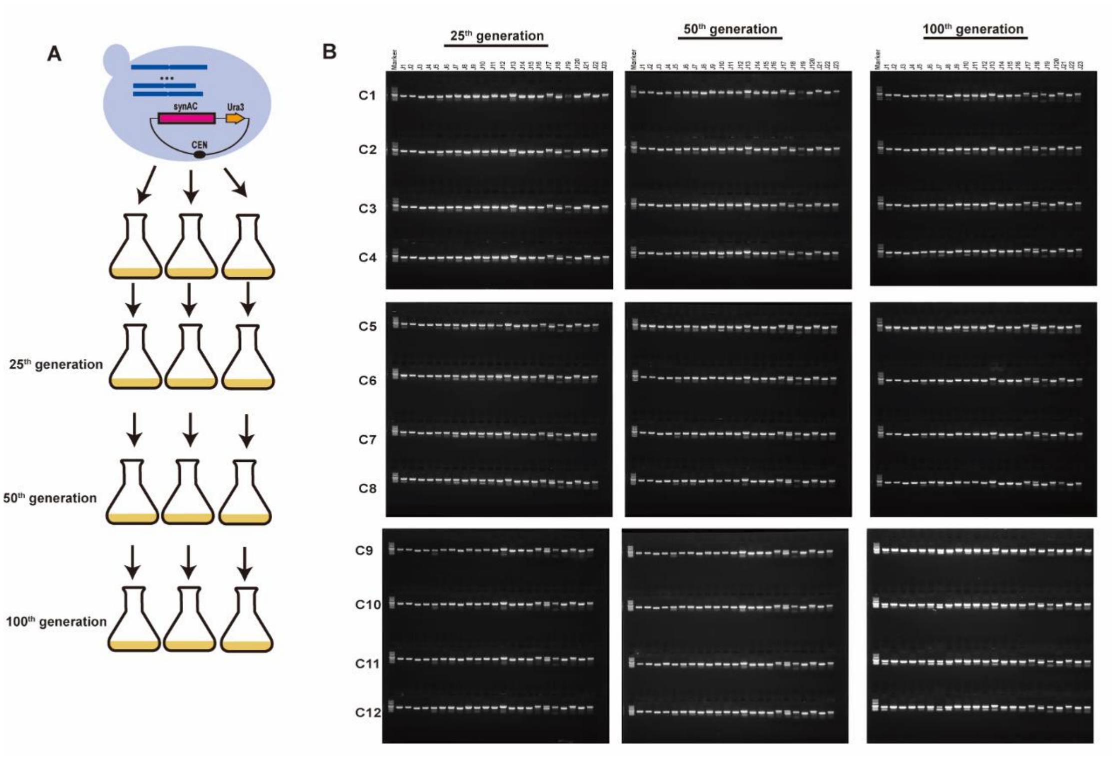

Fig. S9.The stability of synAC in serially passages in the present of selection. A. The process of serially passages. **B.** Randomly selected 12 individual colonies from solid agar plates and use 23 specific PCR tags to validate the genomic integrity of synAC, after passages 25, 50and 100 generations.
