# Supplementary material for: Convenient synthesis and delivery of a megabase-scale designer accessory chromosome empower biosynthetic capacity
Source: Cell Res. 2024 Feb 8;34(4):309–22. doi: 10.1038/s41422-024-00934-3 (PMC10978979; doi:10.1038/s41422-024-00934-3)
Supplement: Supplementary file 12 — Supplementary information, Fig. S12 [file 41422_2024_934_MOESM12_ESM.pdf]

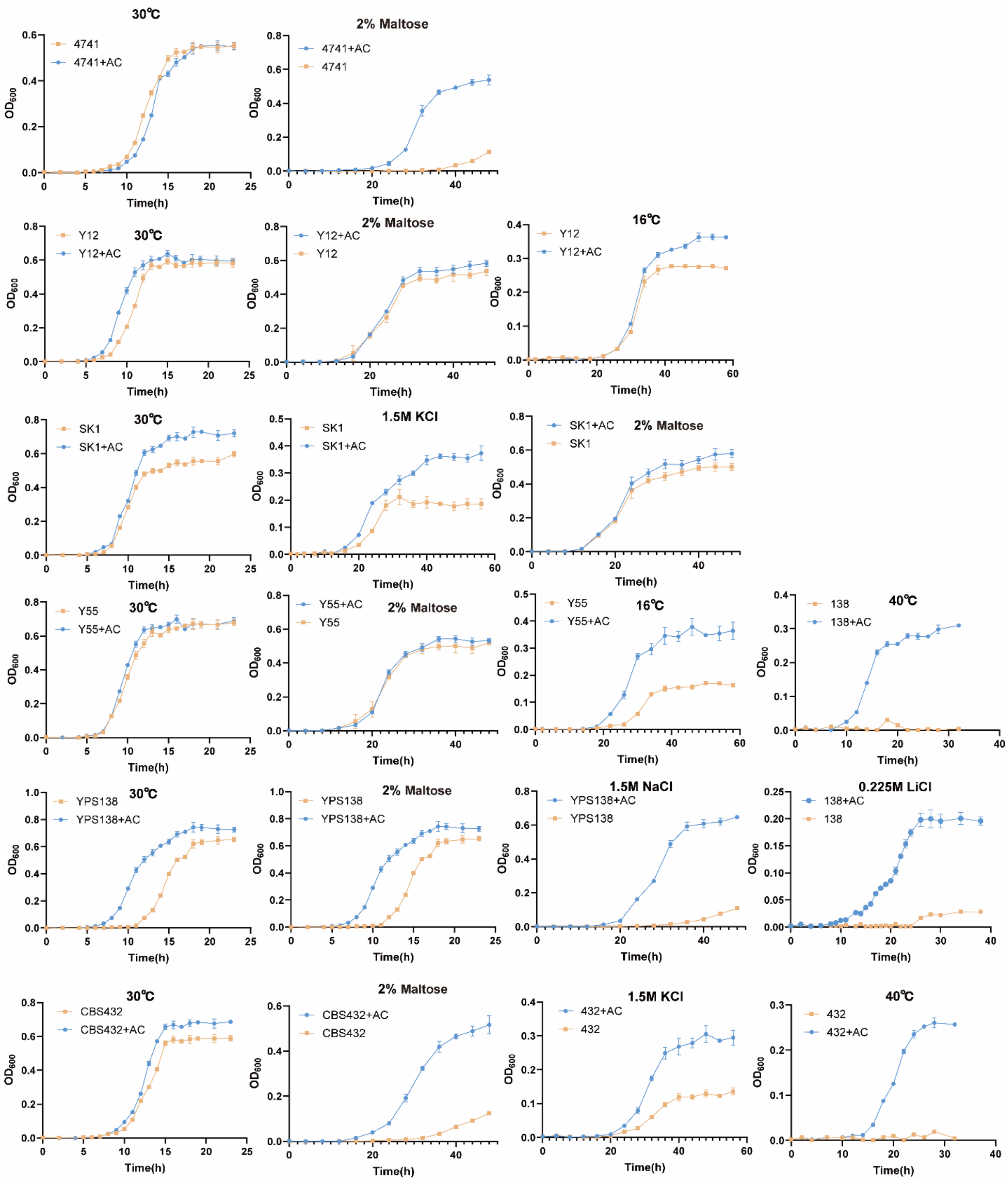

Fig. S12. Growth curves of the strains contain synAC compared to the wild strain in various conditions. Data are presented as mean values +/− SD, based upon values obtained from three independent biological replicates.
