# Supplementary material for: Convenient synthesis and delivery of a megabase-scale designer accessory chromosome empower biosynthetic capacity
Source: Cell Res. 2024 Feb 8;34(4):309–22. doi: 10.1038/s41422-024-00934-3 (PMC10978979; doi:10.1038/s41422-024-00934-3)
Supplement: Supplementary file 13 — Supplementary information, Fig. S13 [file 41422_2024_934_MOESM13_ESM.pdf]

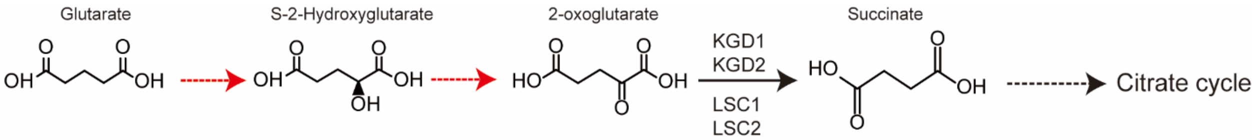


**Fig. S13. The speculation glutarate hydroxylation pathways from glutarate into the citrate cycle in yeast.** The red dotted arrow showed that this reaction was absent in *S. cerevisiae* BY4741.
