# Supplementary material for: Convenient synthesis and delivery of a megabase-scale designer accessory chromosome empower biosynthetic capacity
Source: Cell Res. 2024 Feb 8;34(4):309–22. doi: 10.1038/s41422-024-00934-3 (PMC10978979; doi:10.1038/s41422-024-00934-3)
Supplement: Supplementary file 14 — Supplementary information, Fig. S14 [file 41422_2024_934_MOESM14_ESM.pdf]

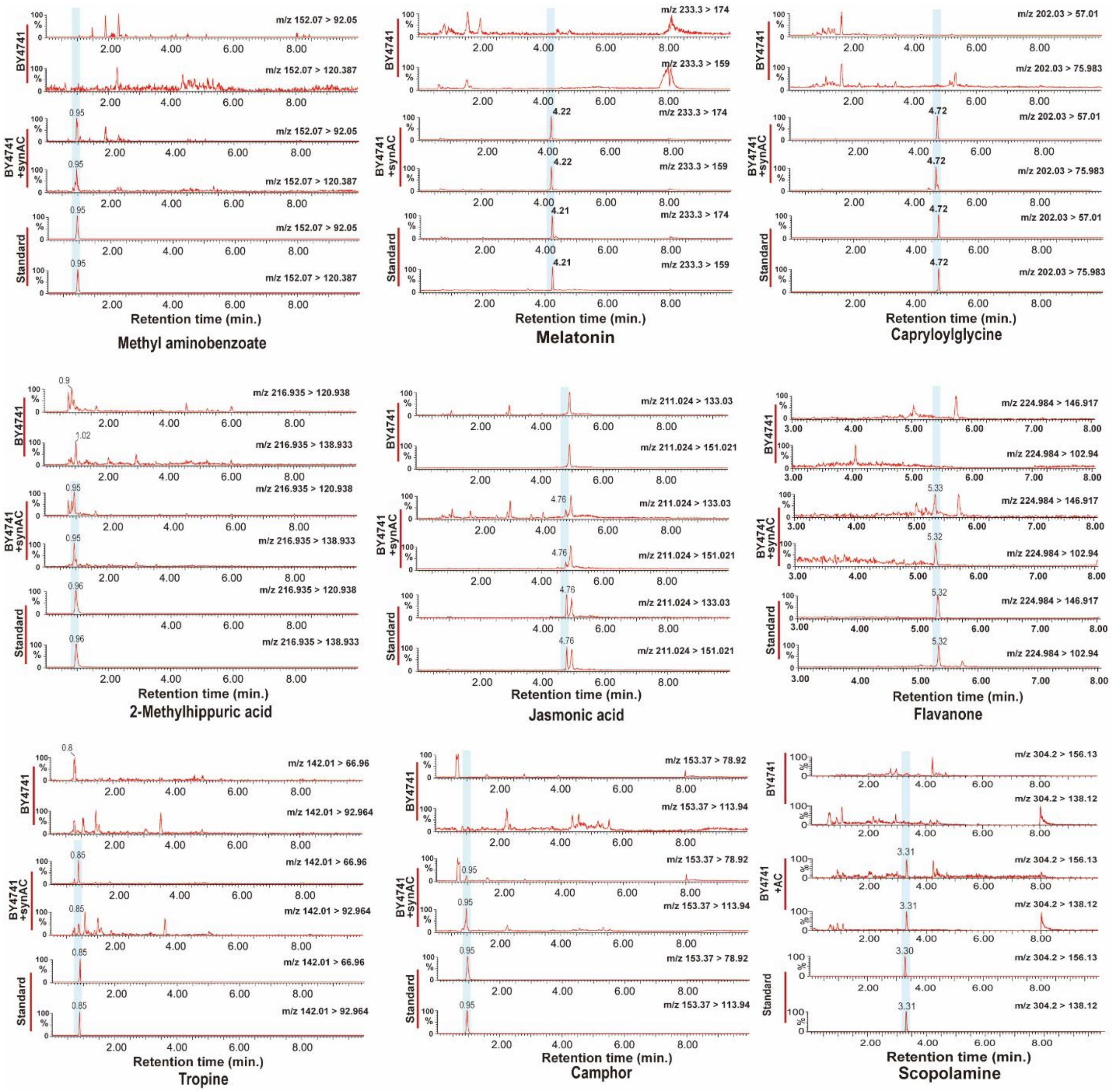

Fig. S14. The identified potential new compounds of the strains harboring synAC using authentic standards. MRM traces are shown for metabolite using the highest precursor ion/product ion transitions. Chromatogram traces are representative of three biological replicates.
