# Supplementary material for: Convenient synthesis and delivery of a megabase-scale designer accessory chromosome empower biosynthetic capacity
Source: Cell Res. 2024 Feb 8;34(4):309–22. doi: 10.1038/s41422-024-00934-3 (PMC10978979; doi:10.1038/s41422-024-00934-3)
Supplement: Supplementary file 15 — Supplementary information, Fig. S15 [file 41422_2024_934_MOESM15_ESM.pdf]

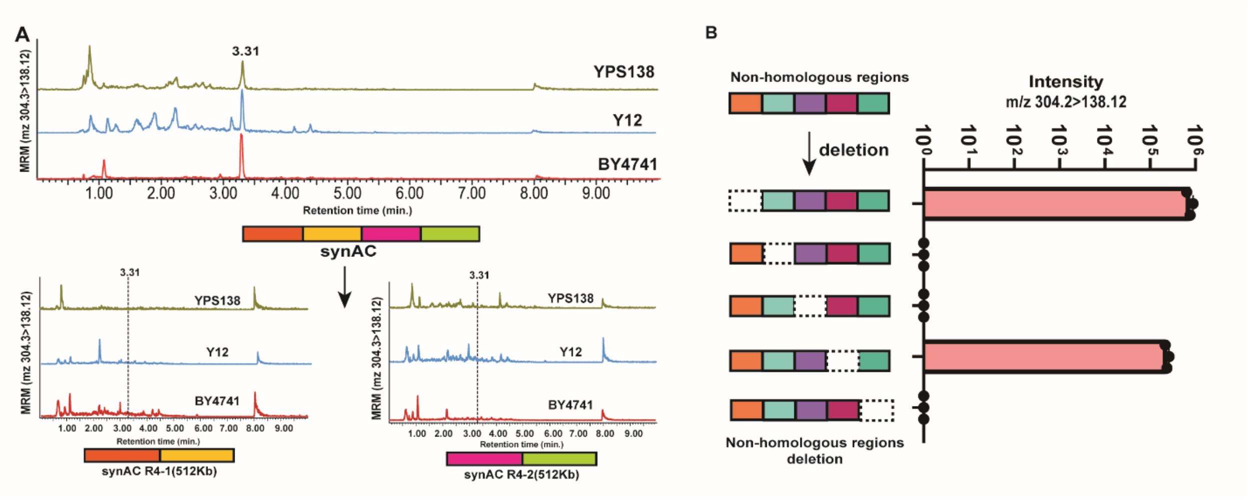

Fig. S15. Testing the production of scopolamine for intermediate assembly strains and partial deletion strains. A. Testing the production of scopolamine in three strains (BY4741, Y12 and YPS138) harboring half of the synAC. The results of MRM showed that neither of the intermediate strains was detected with scopolamine. Chromatogram traces are representative of three biological replicates. **B.** Testing the production of scopolamine in synAC segmental knocked-out strains. Five deleted chromosomal segments (~50 kb) in the nonhomologous gene region of the synAC are indicated as white dotted rectangles. The results of MRM showed that deletion of three out of the five regions lost the capability to produce scopolamine, while the other two reserved. Data are mean ± s.d. of the spectral intensity (n = 3) for the highest precursor ion/product ion transitions in MRM. For A and B, strains were cultured in selective SC-Ura media with 2% dextrose at 30°C for 48 h before LC-MS/MS analysis.
