# Supplementary material for: Convenient synthesis and delivery of a megabase-scale designer accessory chromosome empower biosynthetic capacity
Source: Cell Res. 2024 Feb 8;34(4):309–22. doi: 10.1038/s41422-024-00934-3 (PMC10978979; doi:10.1038/s41422-024-00934-3)
Supplement: Supplementary file 16 — Supplementary information, Table S1 [file 41422_2024_934_MOESM16_ESM.pdf]

Table S1. Comparison of large DNA assembly methods

Assembly Methods	Number of positive colonies (per 10^7 cells)	The efficiency of assembly	Time spent of each assembly	Iterative schemes	The Recipient of assembled DNA	Main manipulation
**BASIS**	**~10^2**	**8.5~100%**	**~2d**	**Stepwise**	**Stepwise**	***E. coli* conjugative**
**CAGE**	**<10^3**	**NA**	**~7d**	**Parallel**	***E. coli* genome**	***E. coli* conjugative**
**SwAP-In**	**<10^3**	**0.5%~59%**	**~5-7d**	**Stepwise**	***S. cerevisiae* genome or Plasmid**	**LiAc/ssDNA transformation, Reprint**
**MRA**	**<10^3**	**NA**	**~10-15d**	**Parallel**	***S. cerevisiae* genome**	**LiAc/ssDNA transformation, Sporulation**
**Protoplast transformation**	**<10^3**	**2%**	**~5-7d**	**Parallel**	**Plasmid**	**100 kb fragment purification and enrichment *in vitro*, Protoplast transformation**
**CasHRA**	**<10^2**	**63%~75%**	**~9d**	**Parallel**	**Plasmid**	**Protoplast preparation, Protoplast fusion, LiAc/ssDNA transformation**
**YLC**	**~10^4**	**67~100%**	**~11d**	**Parallel**	**Plasmid**	**Yeast mating, Sporulation**
**HAnDy**	**>10^3**	**60%~80%**	**~3-5d**	**Parallel**	**Plasmid**	**Yeast Mating**
